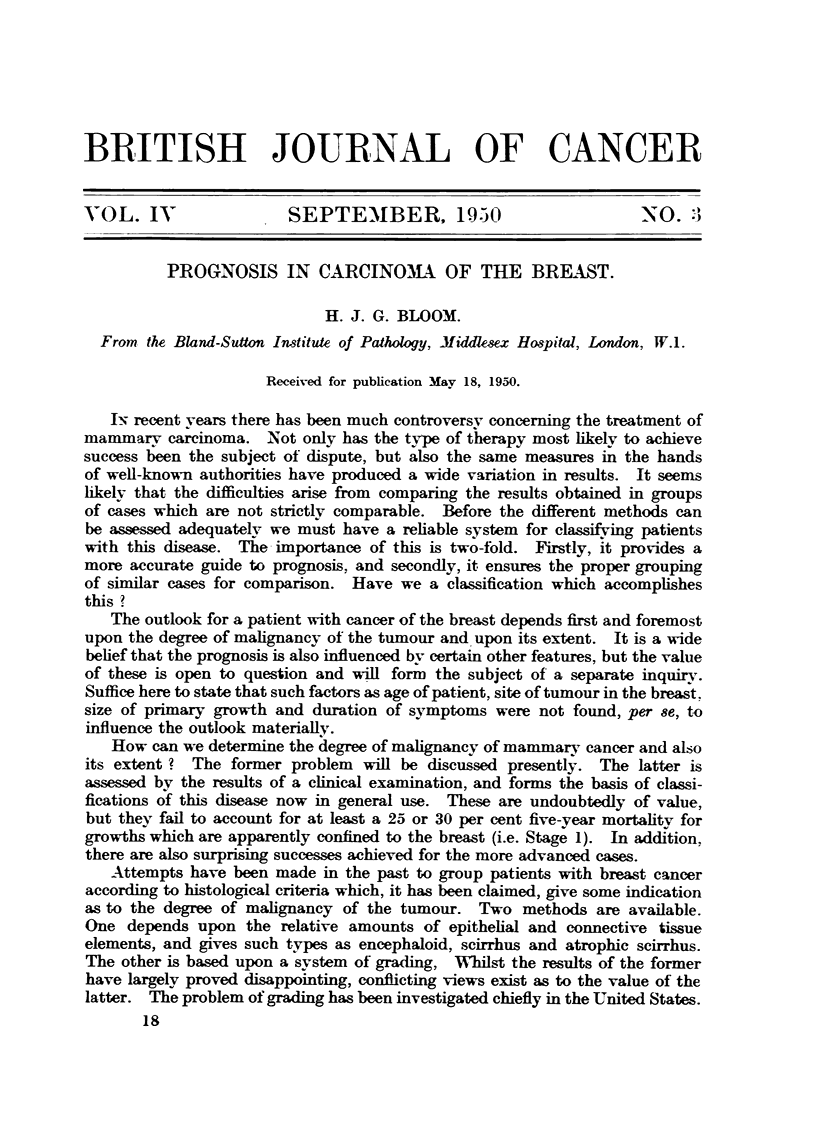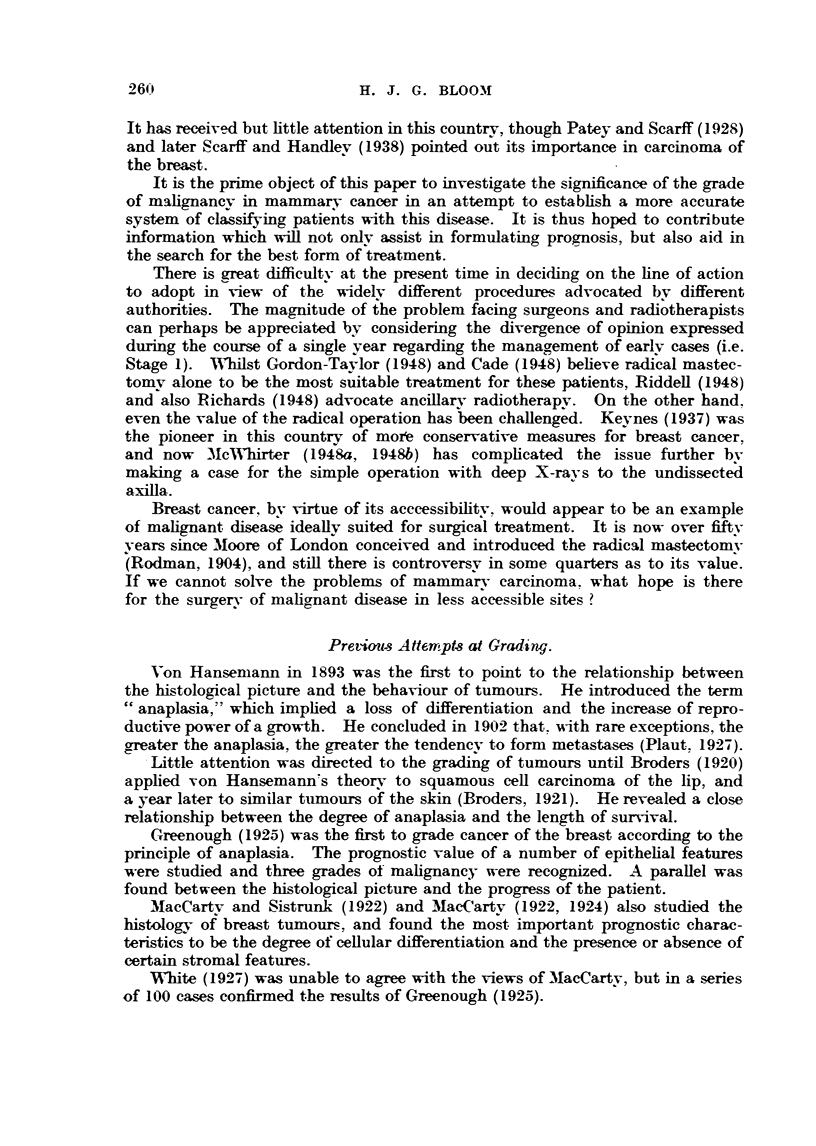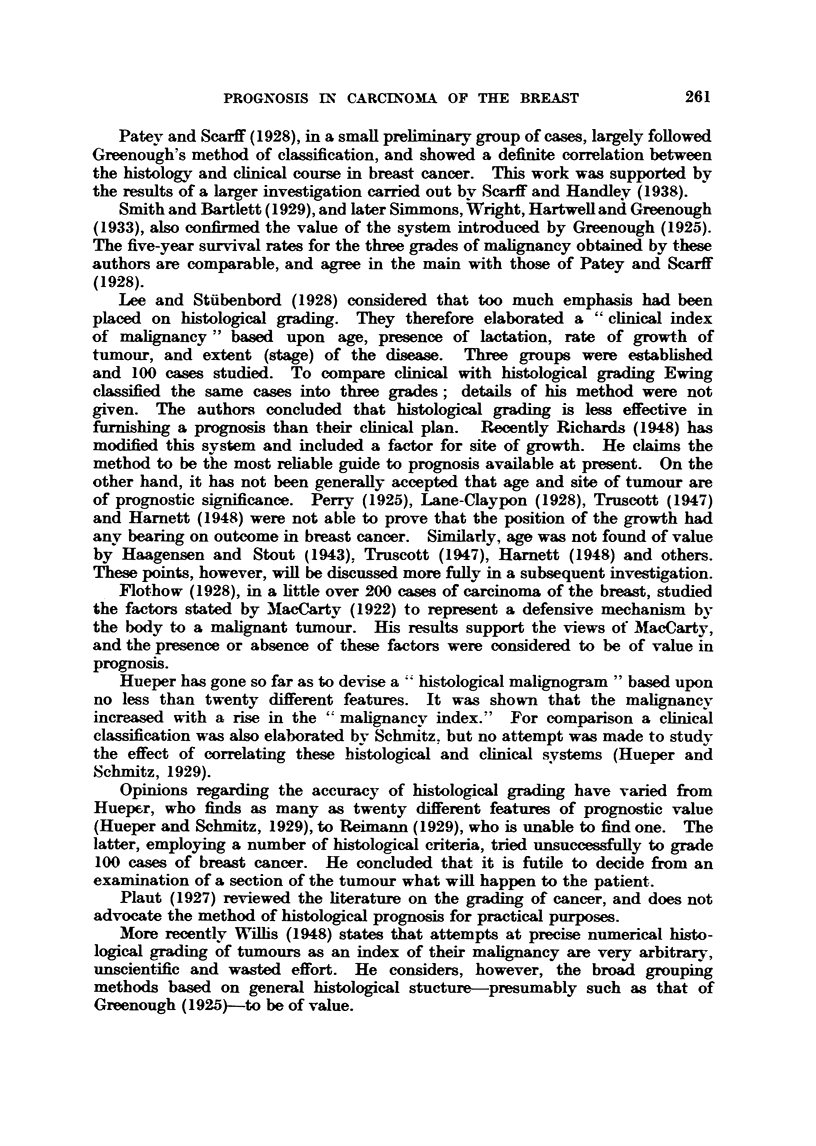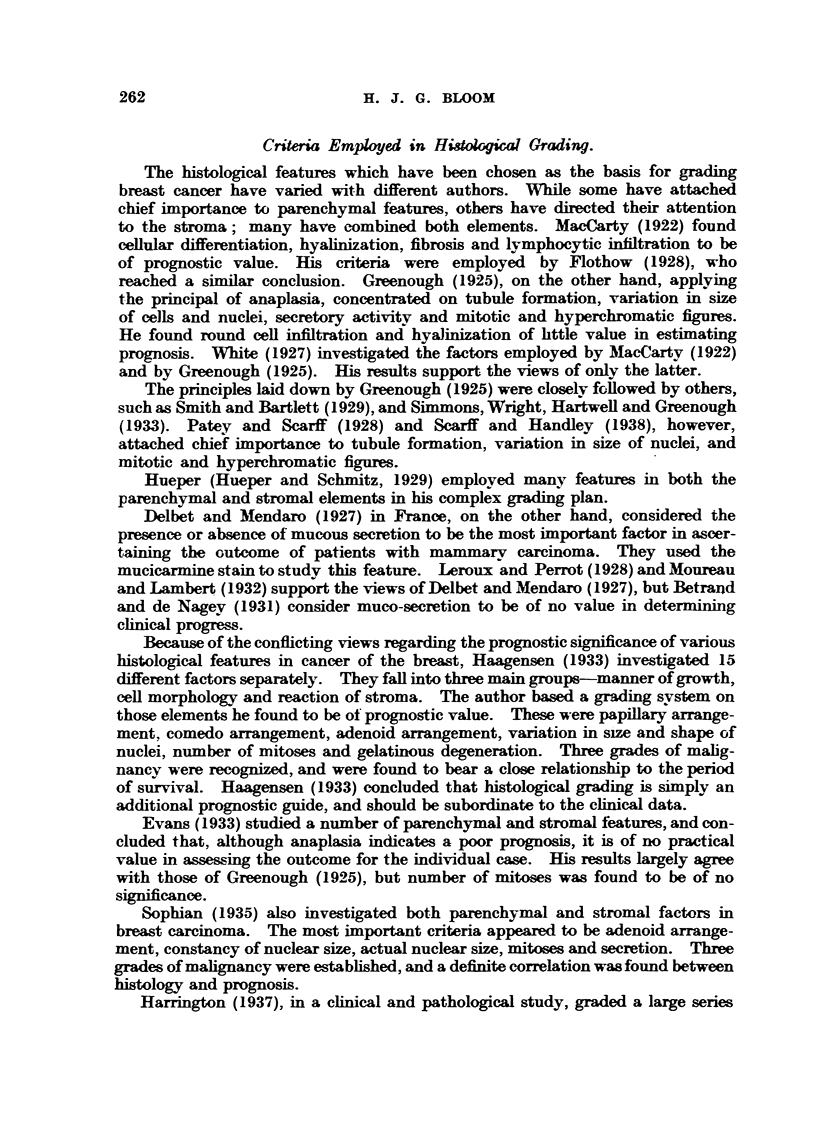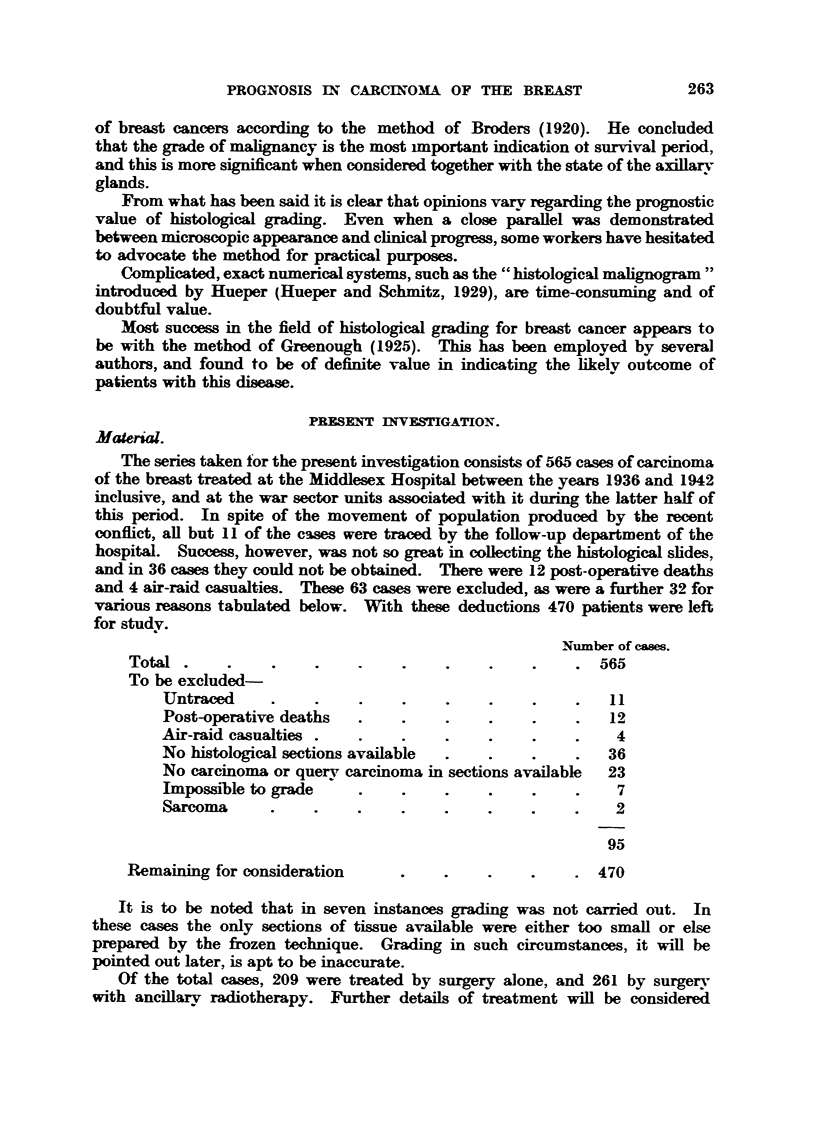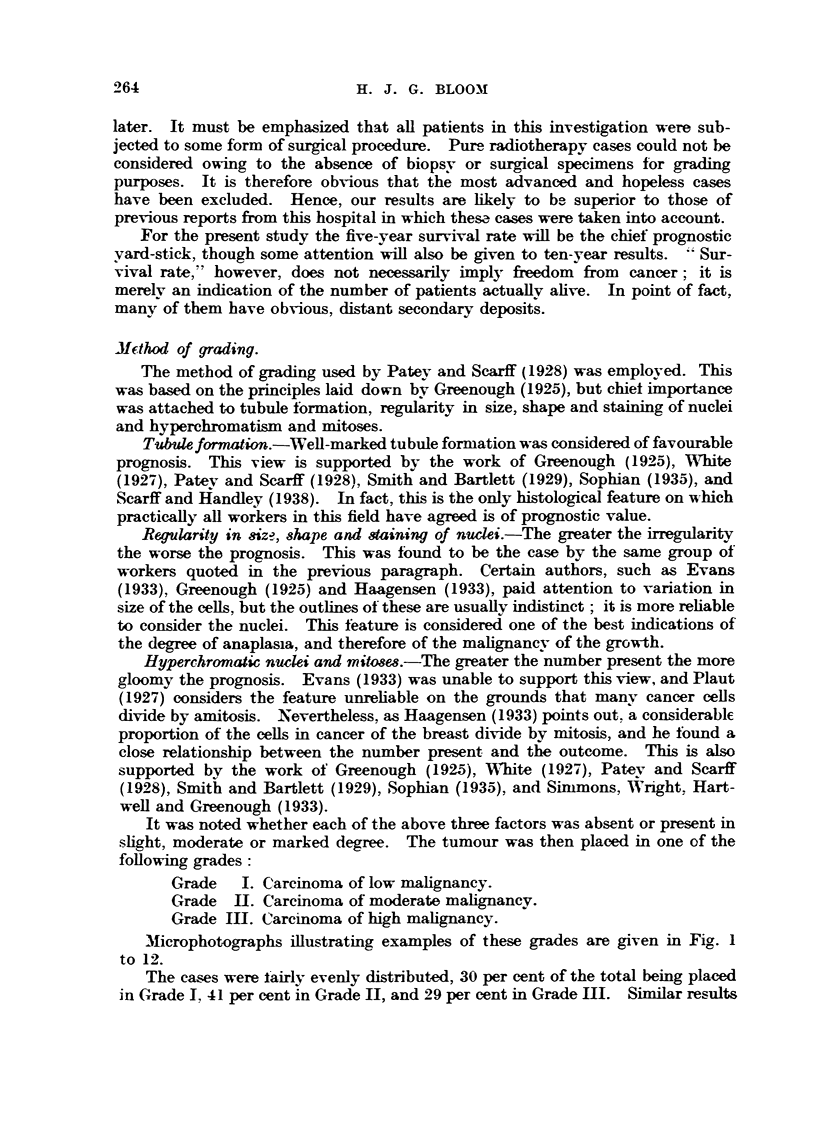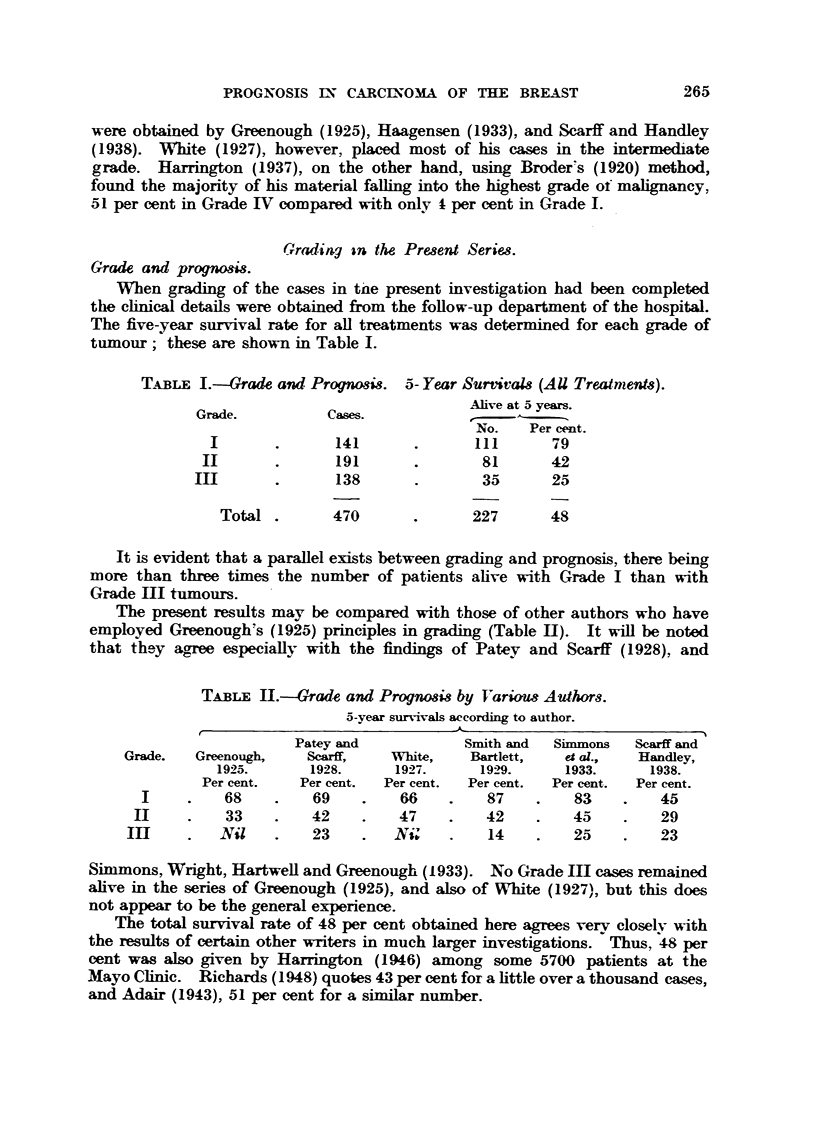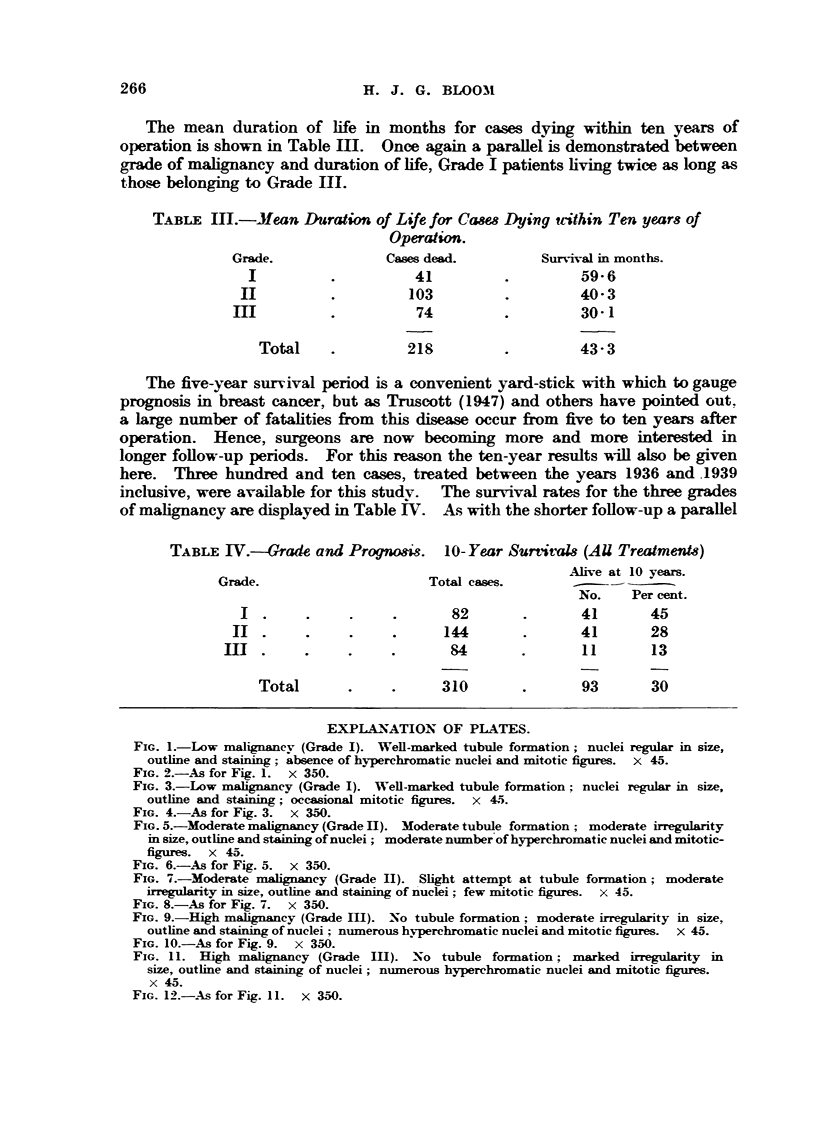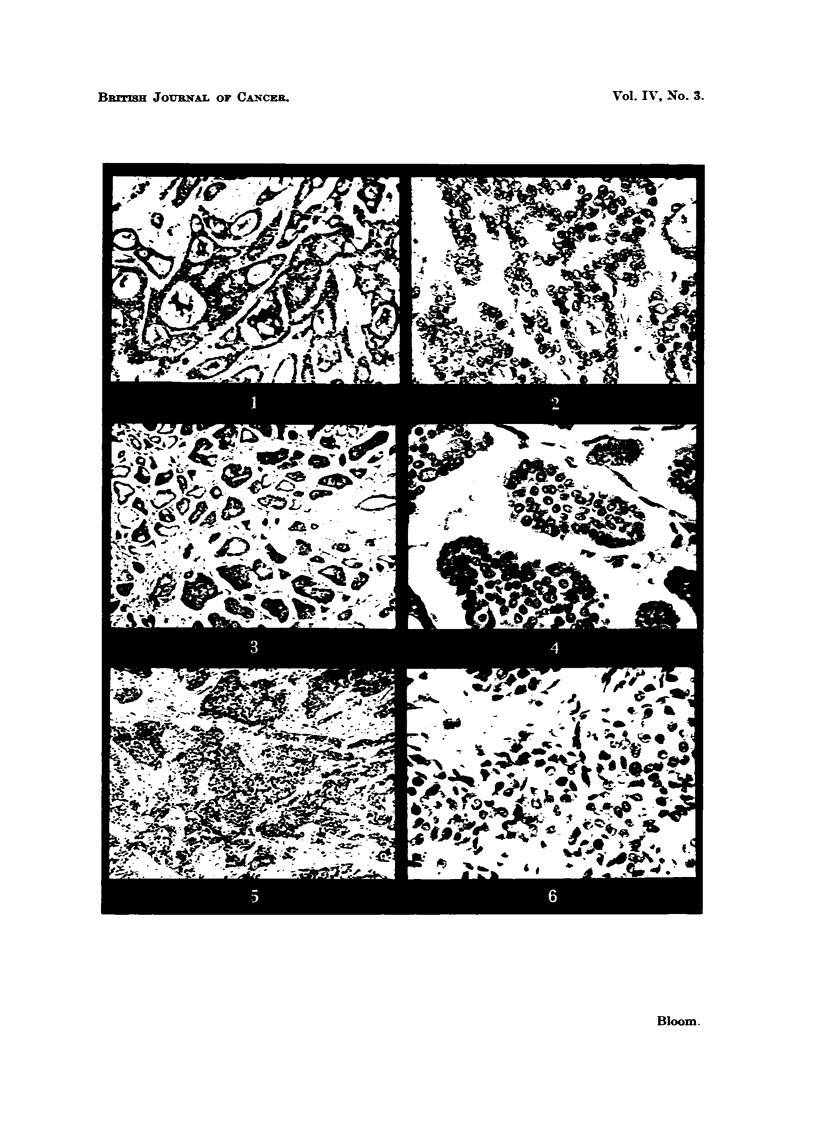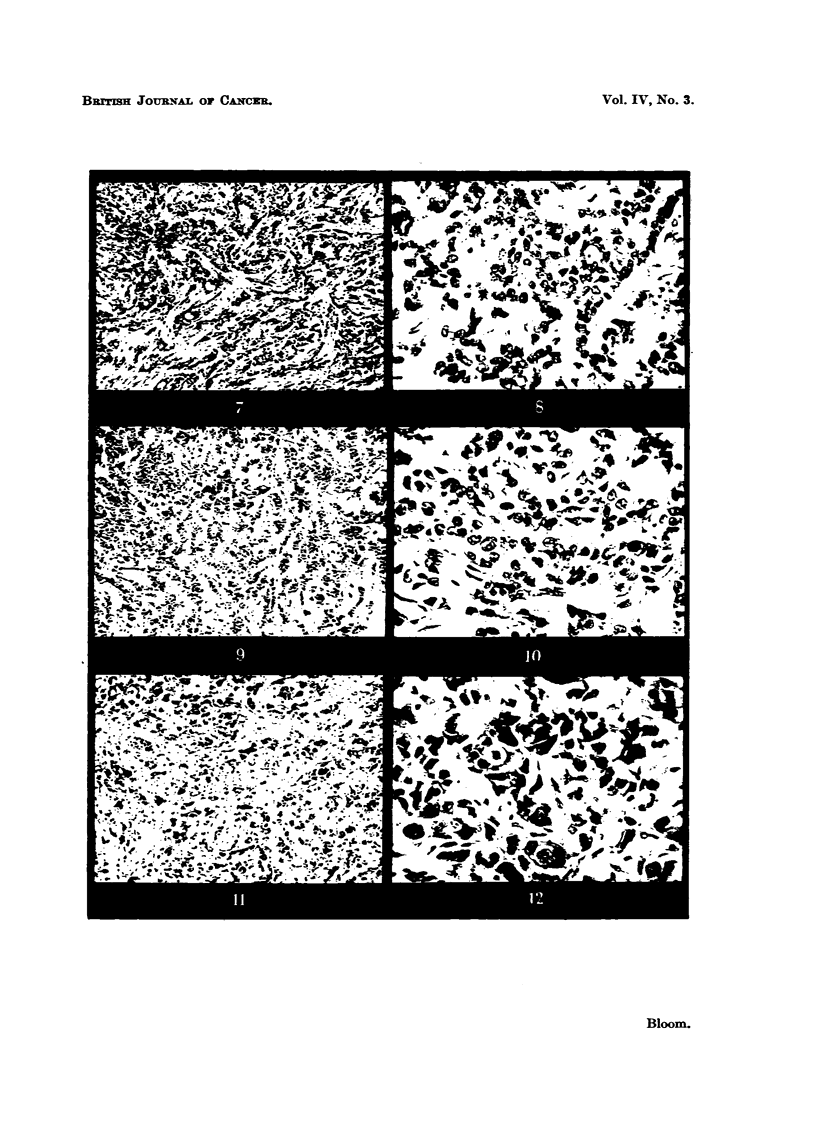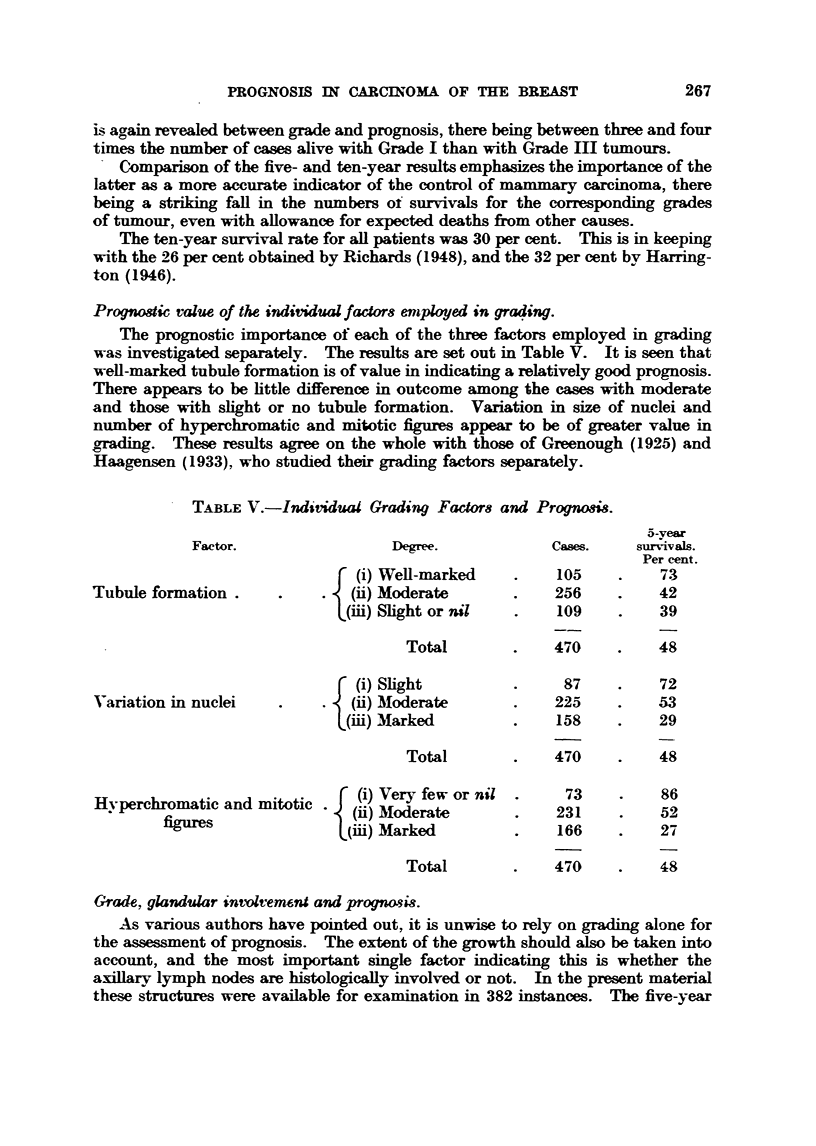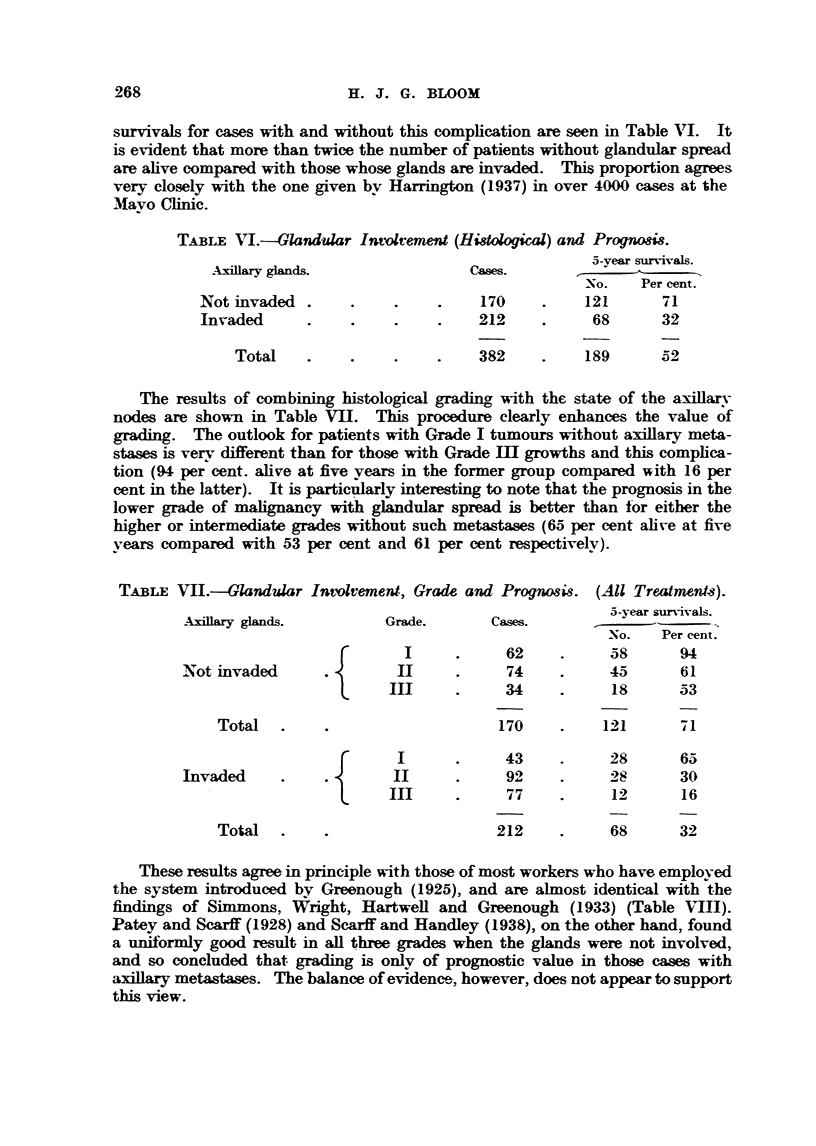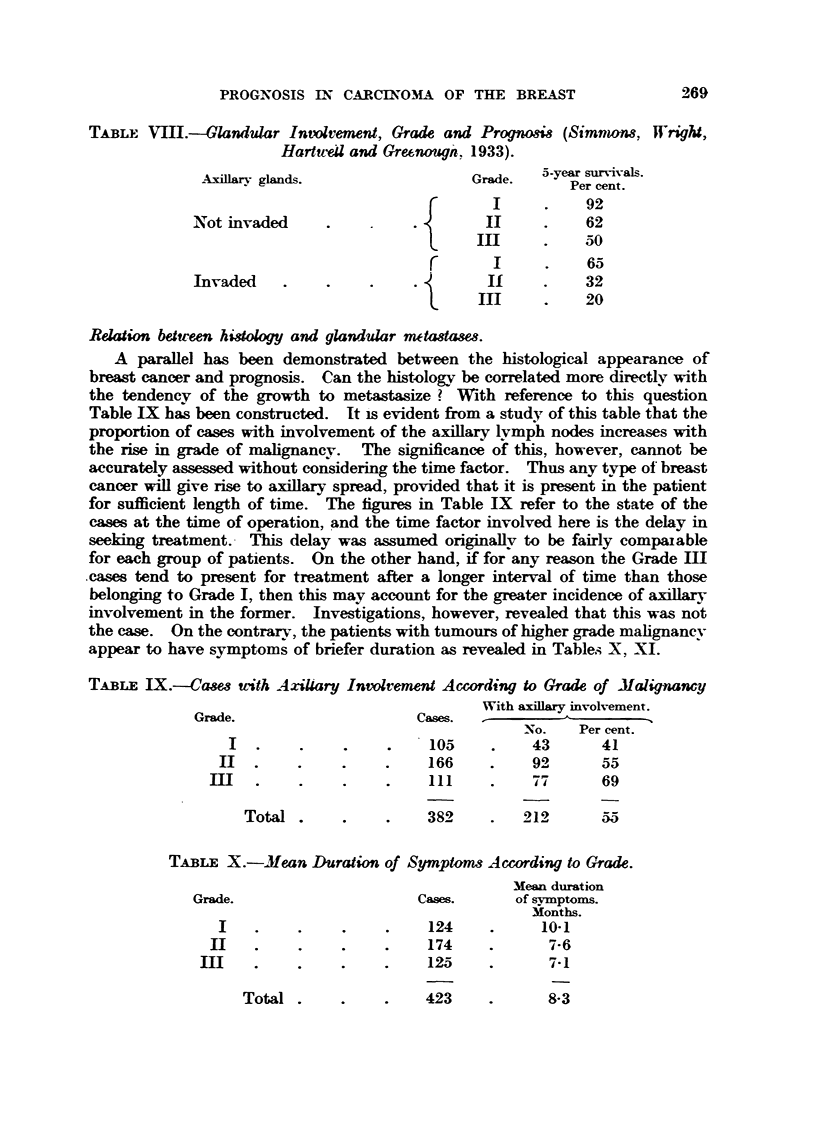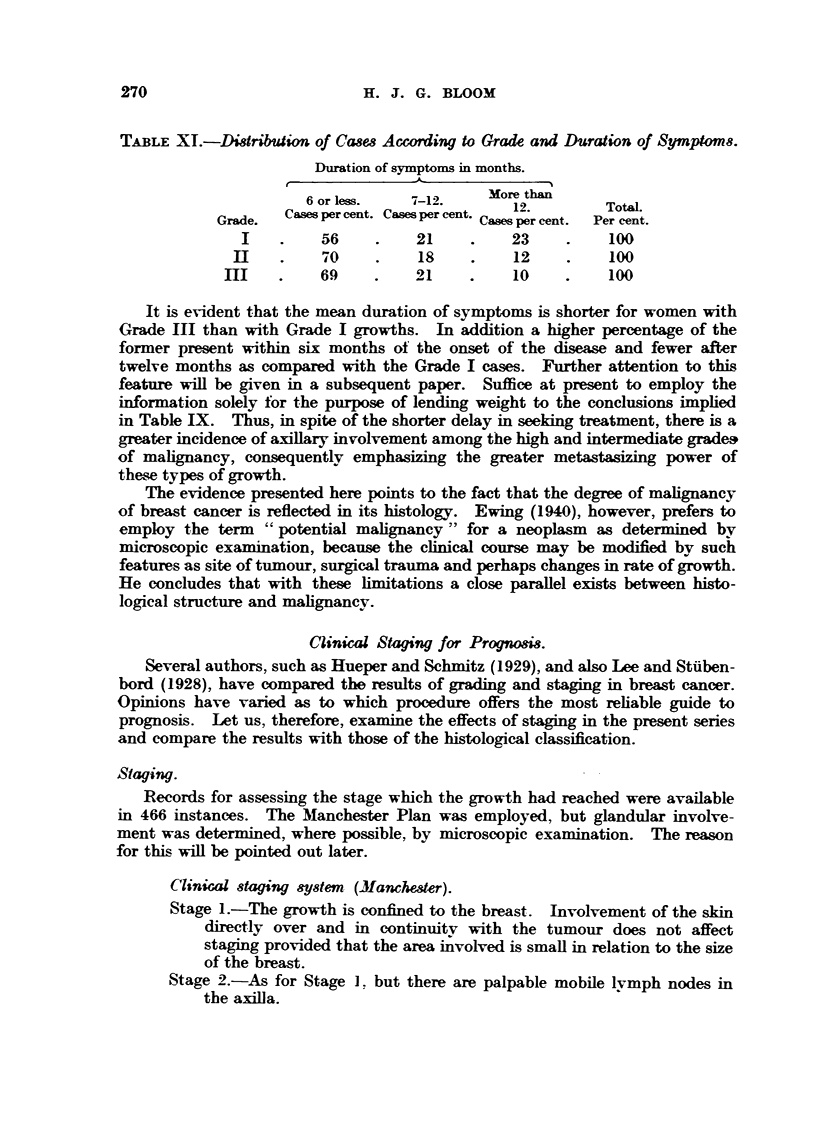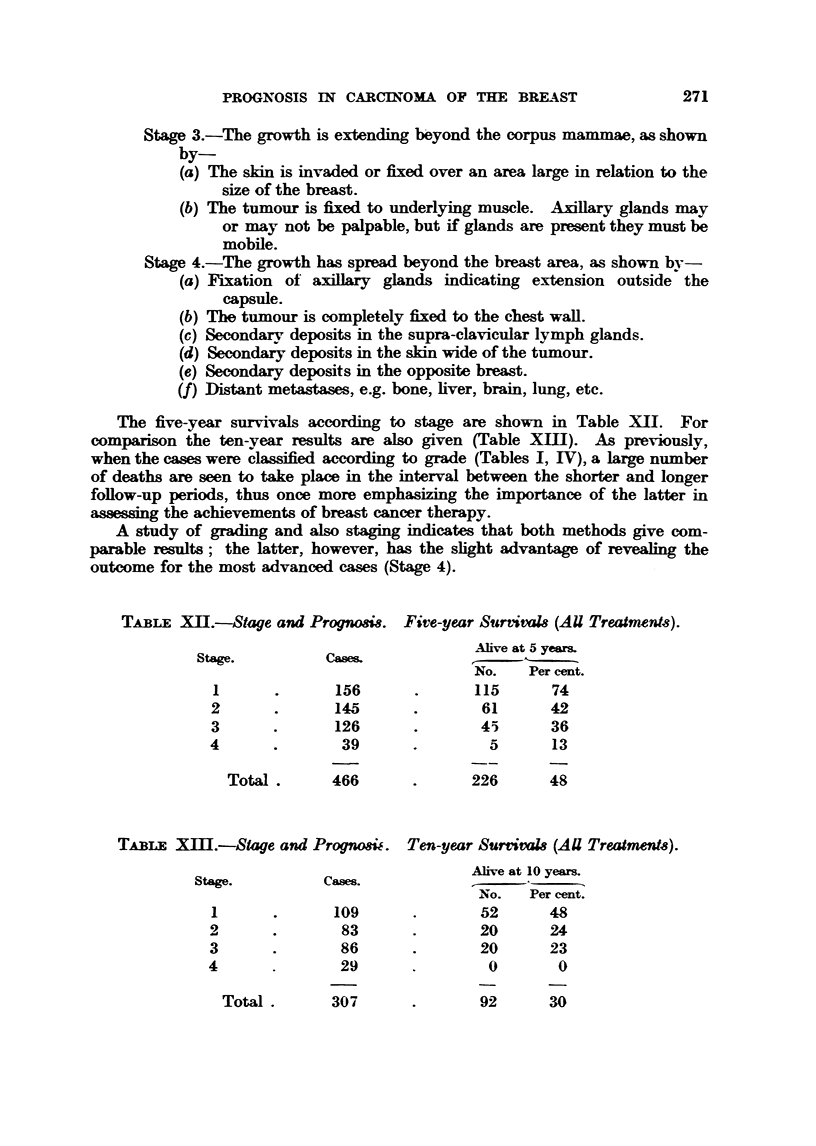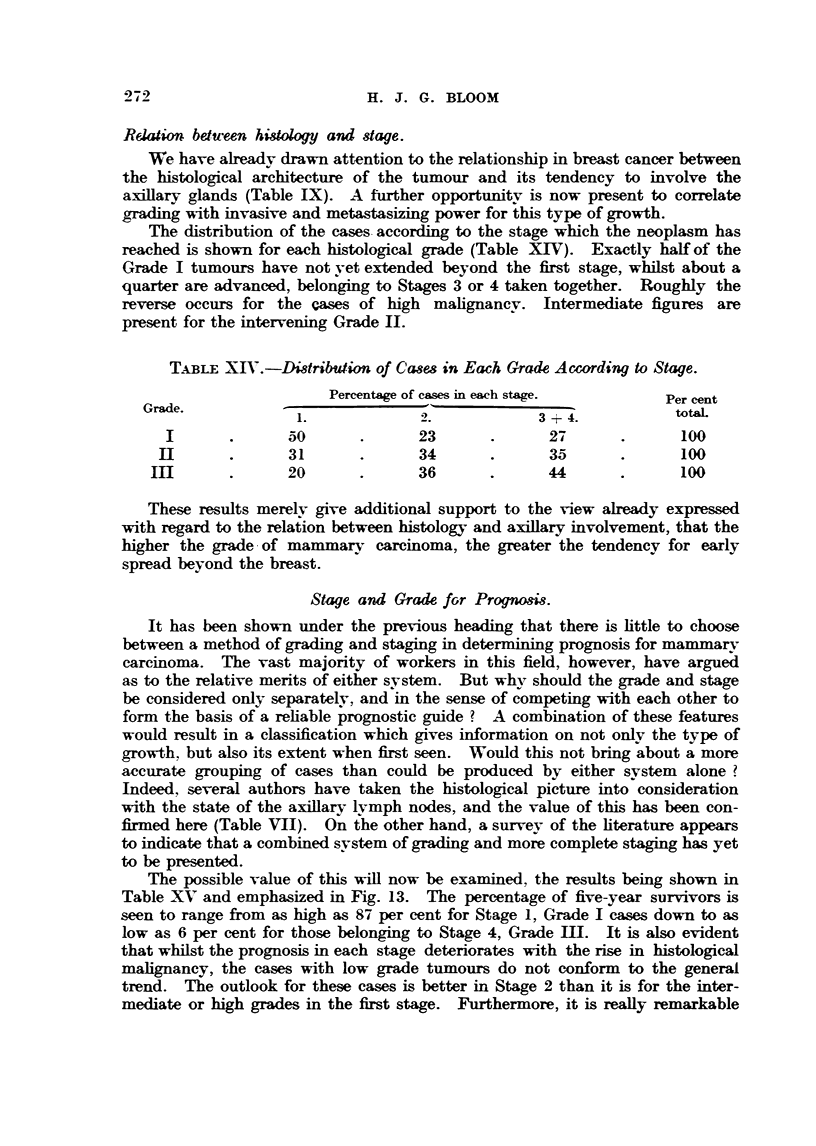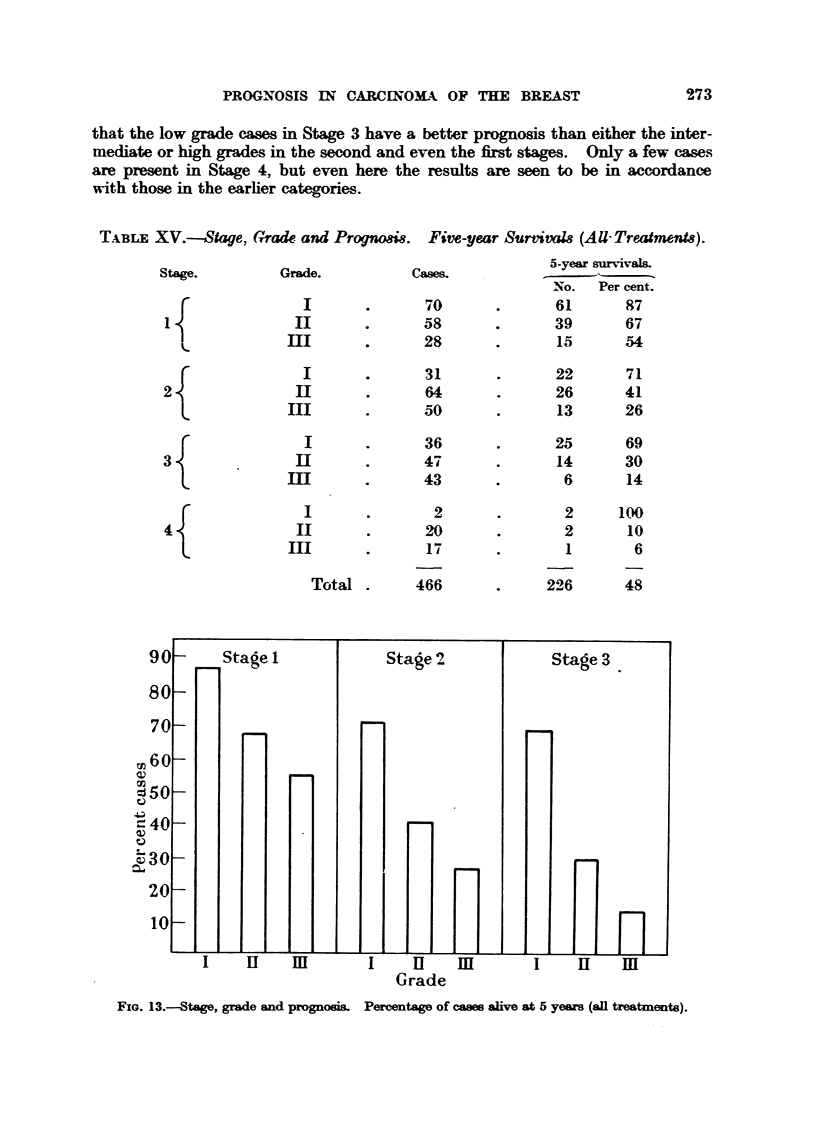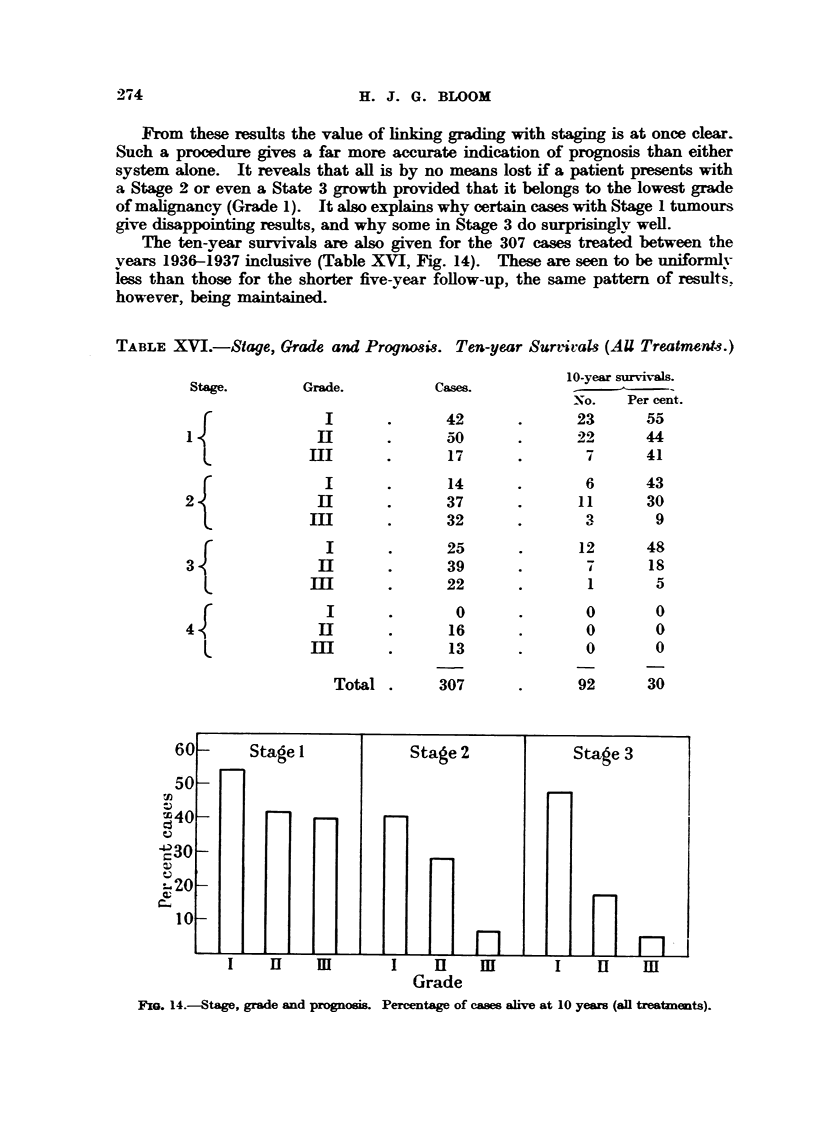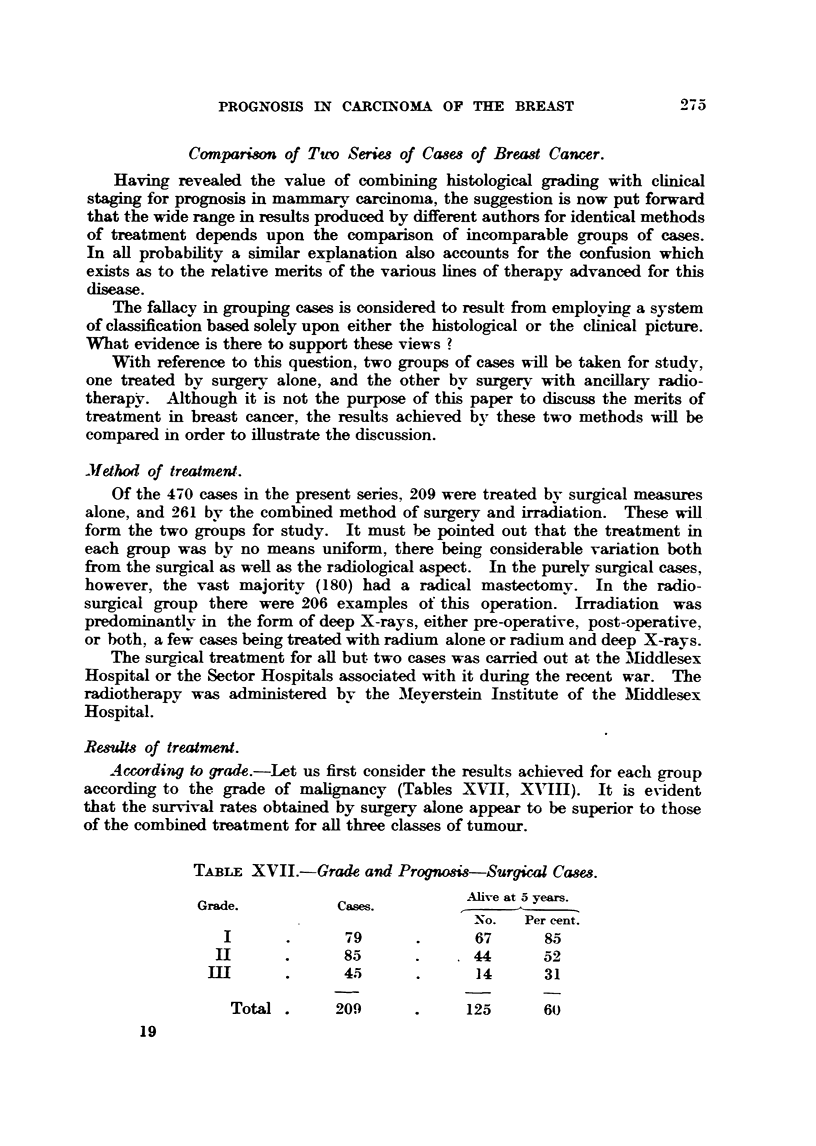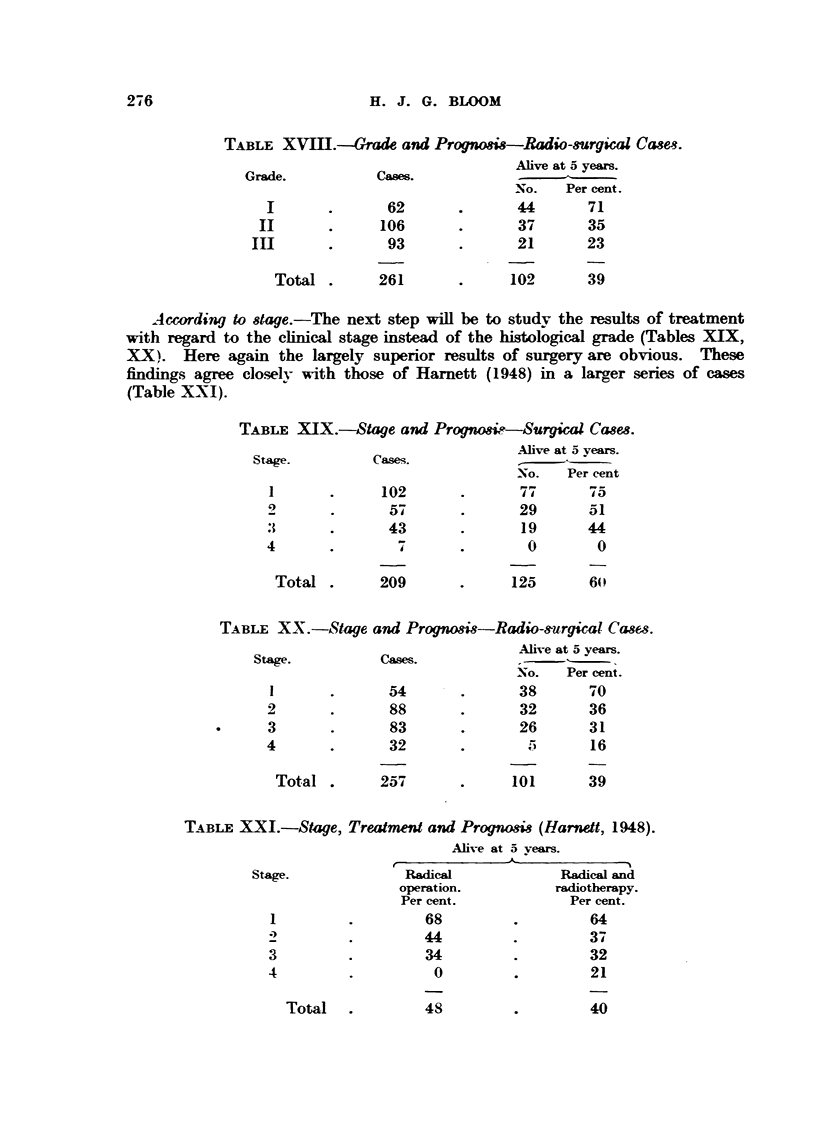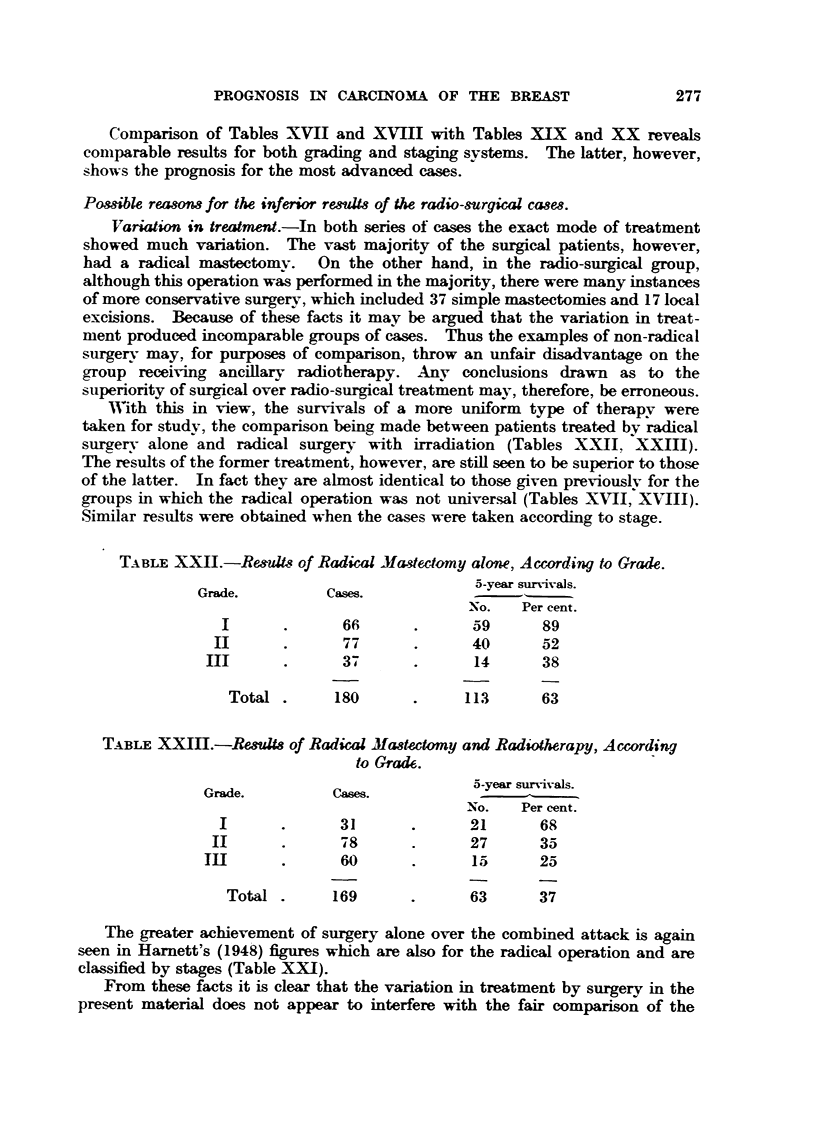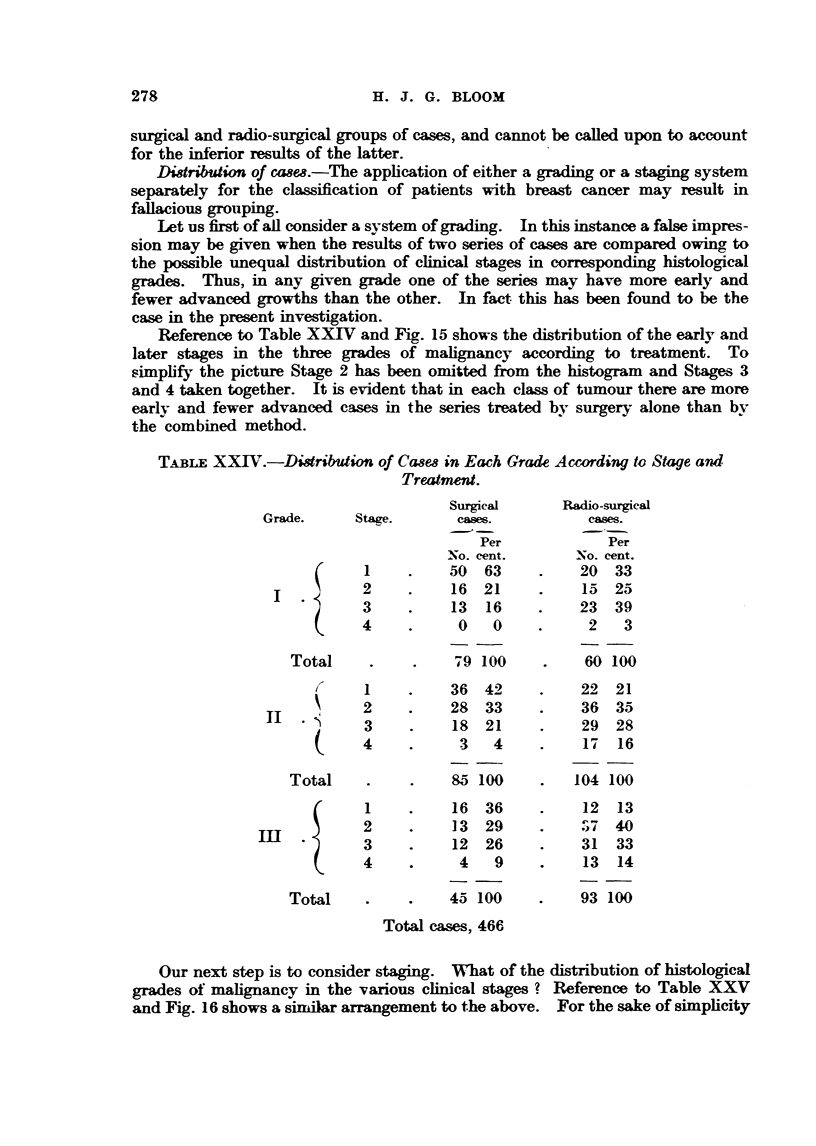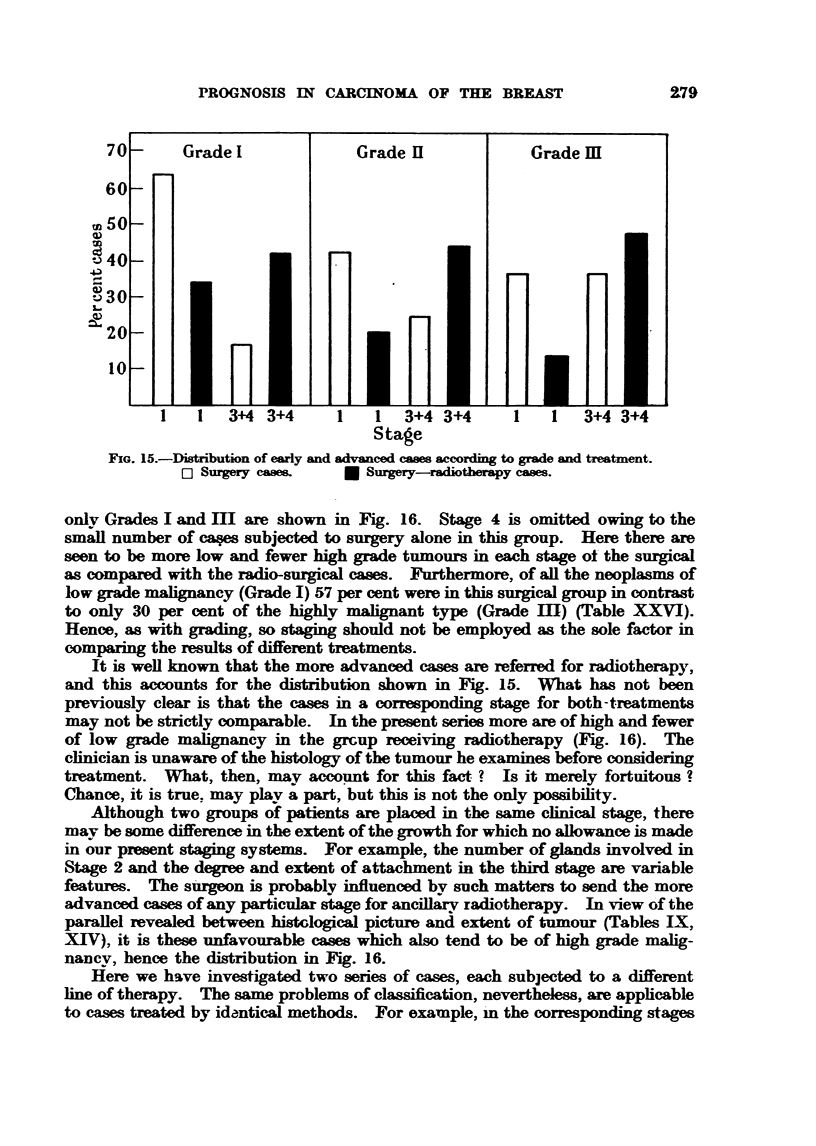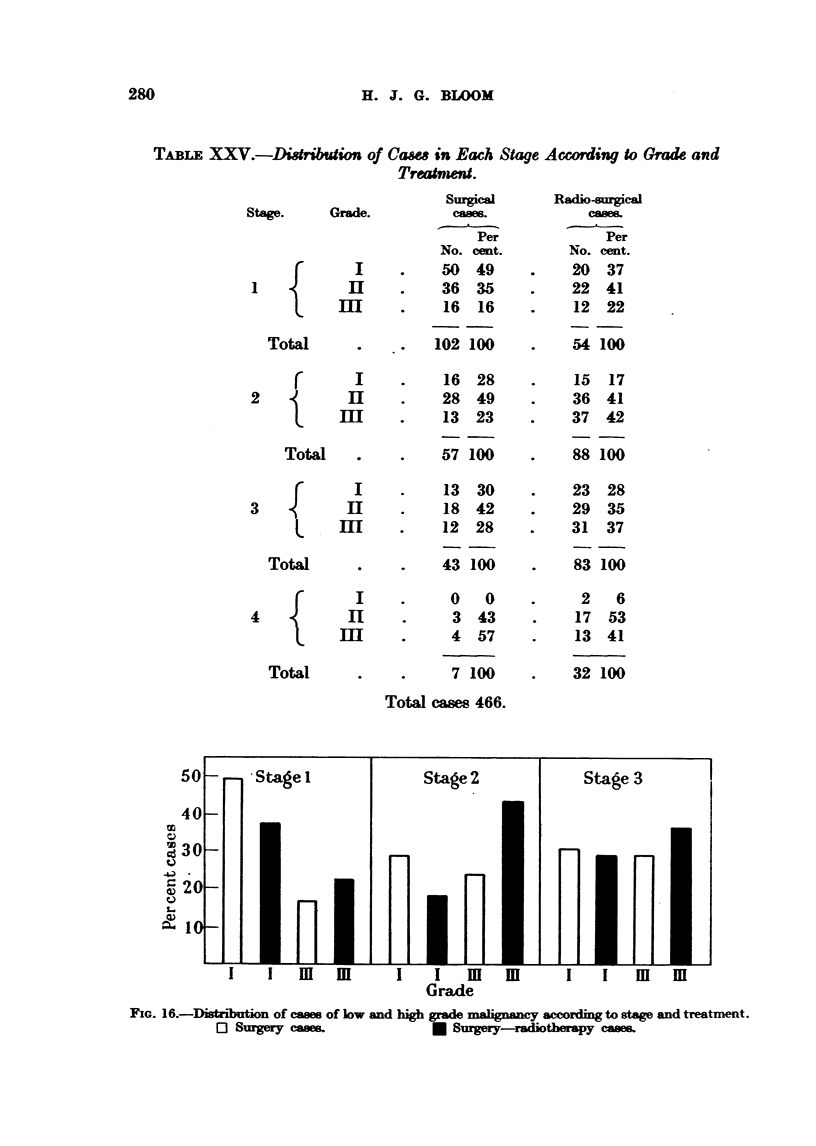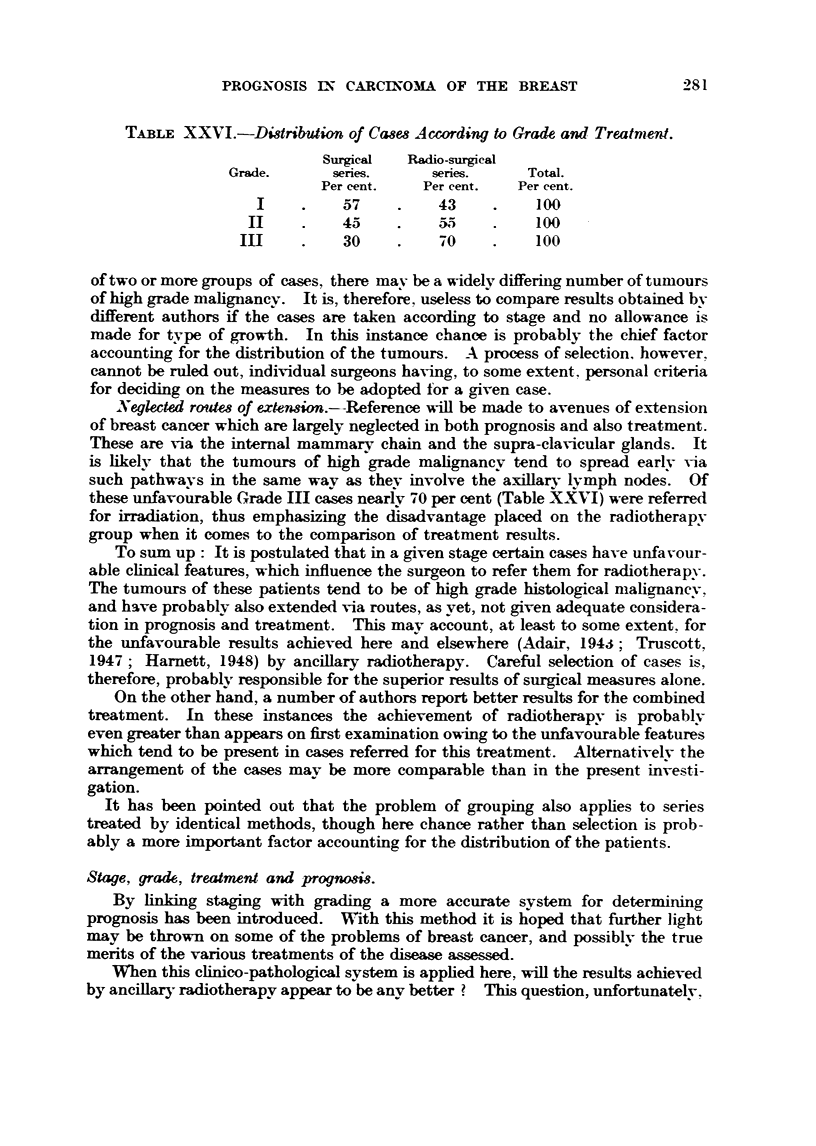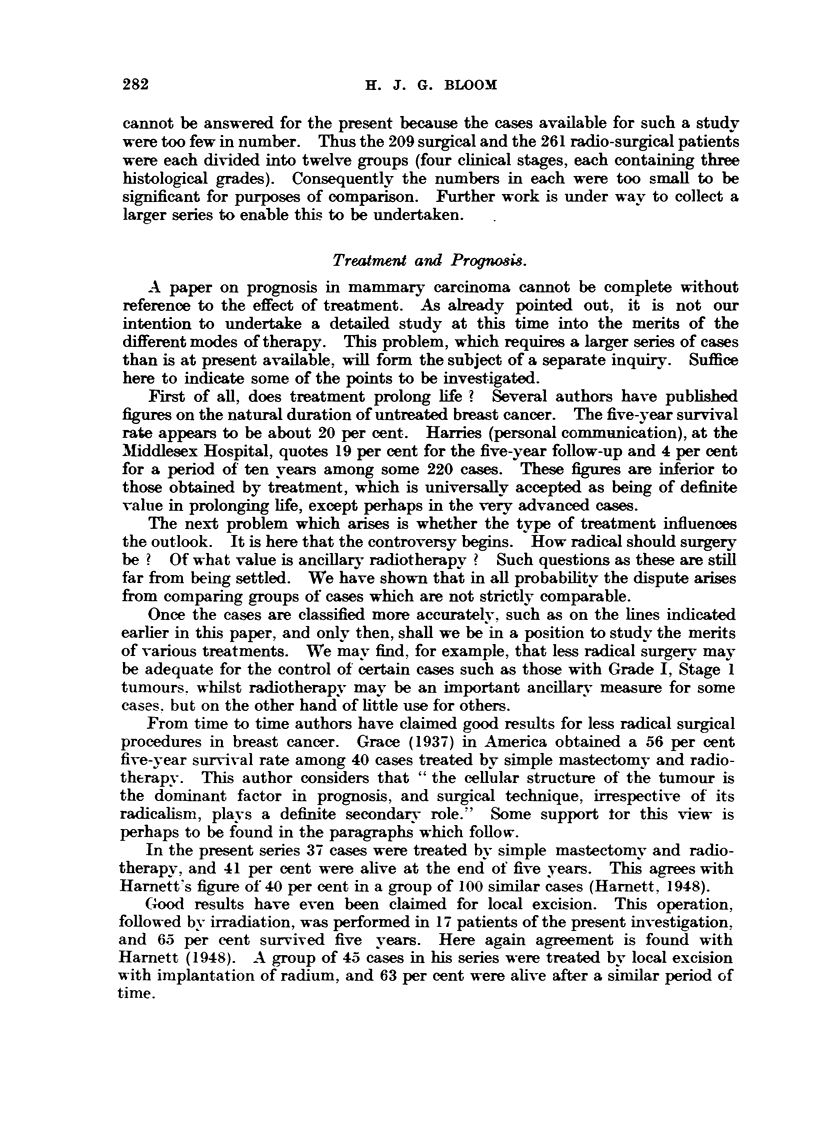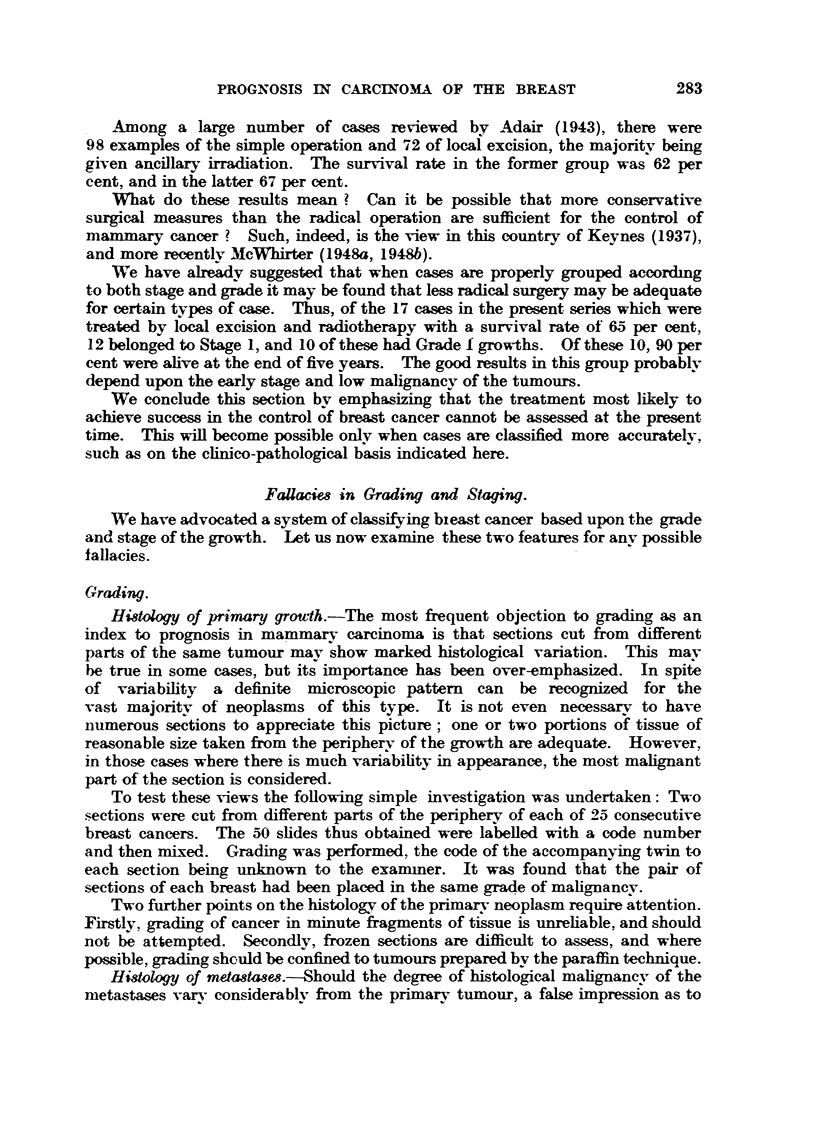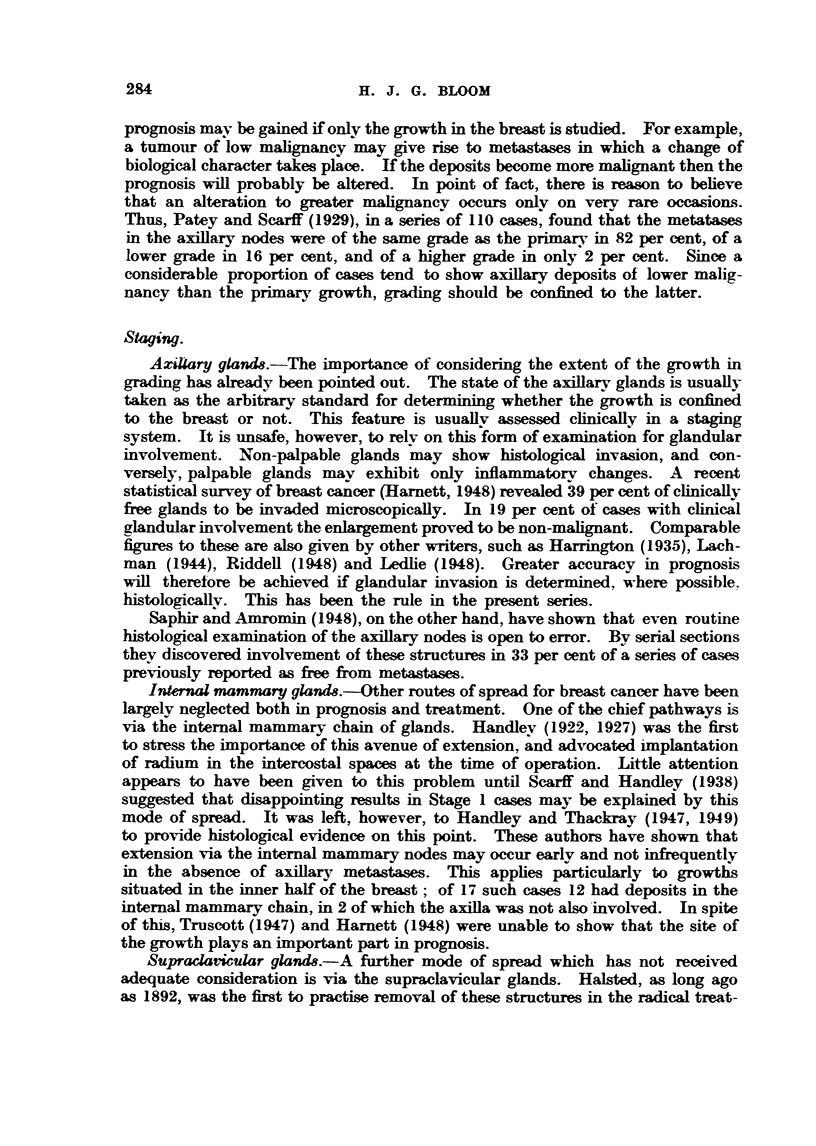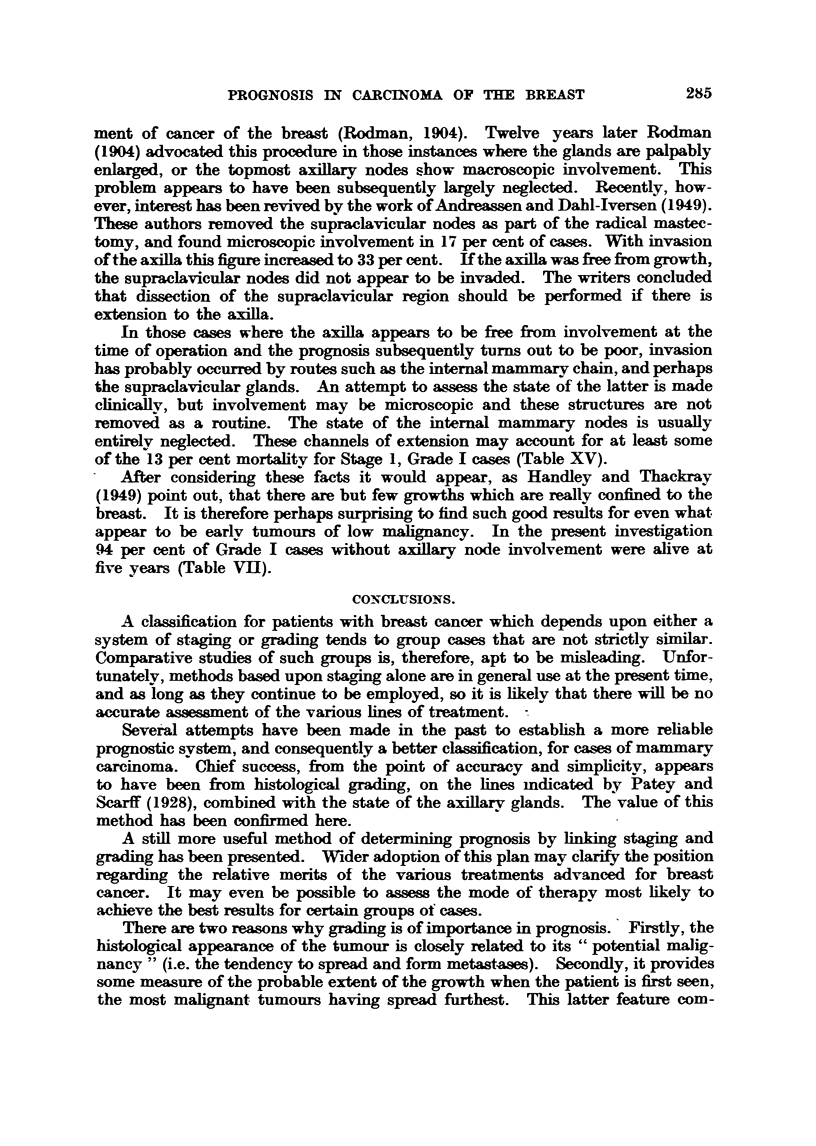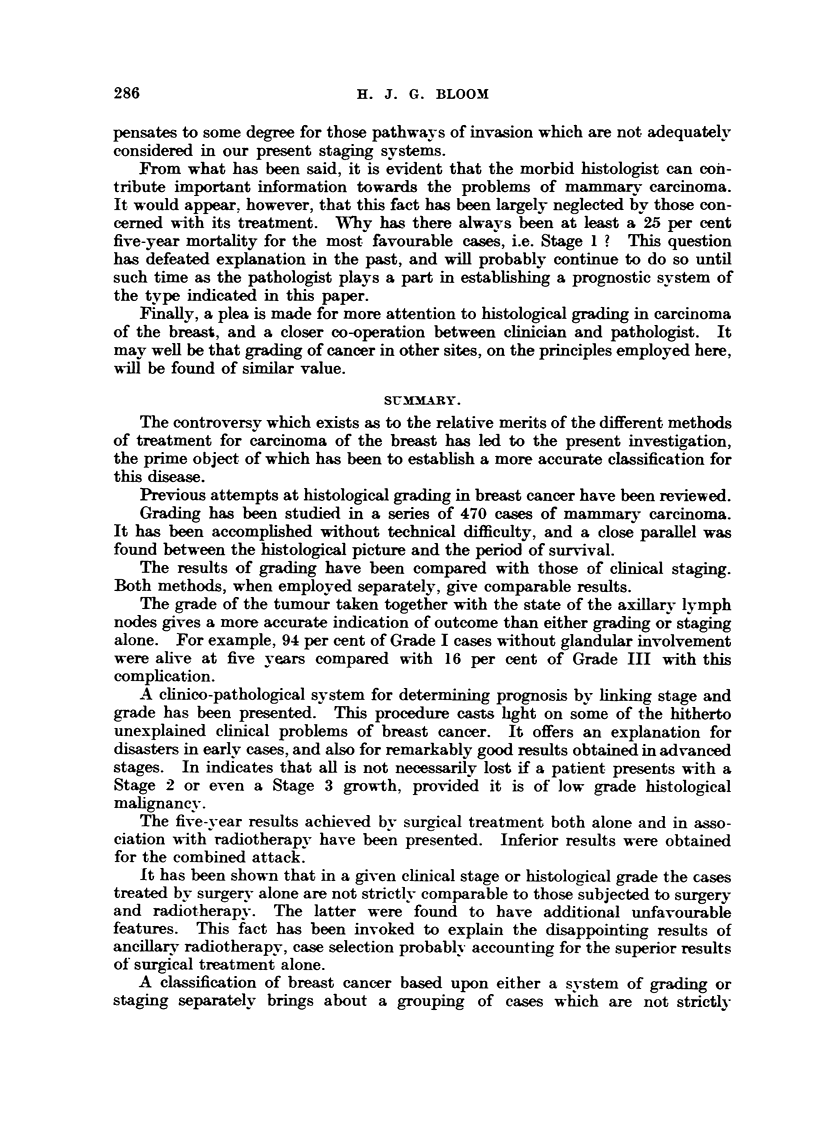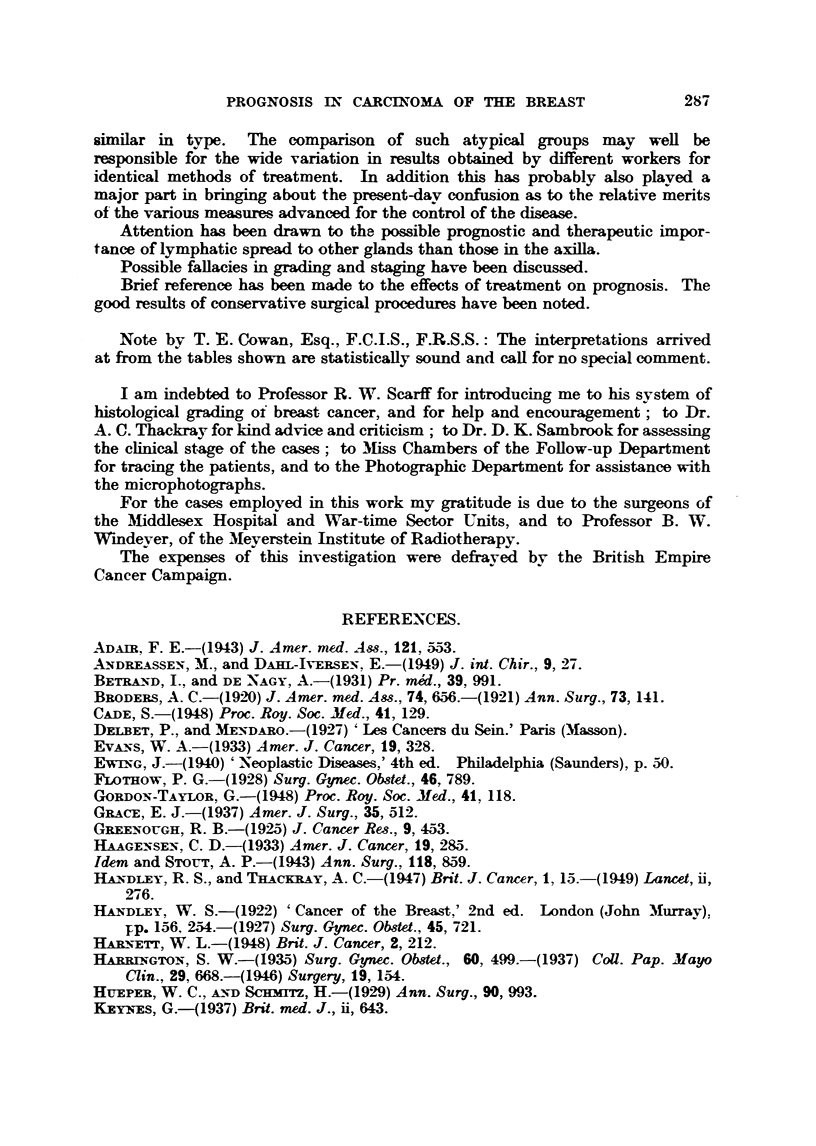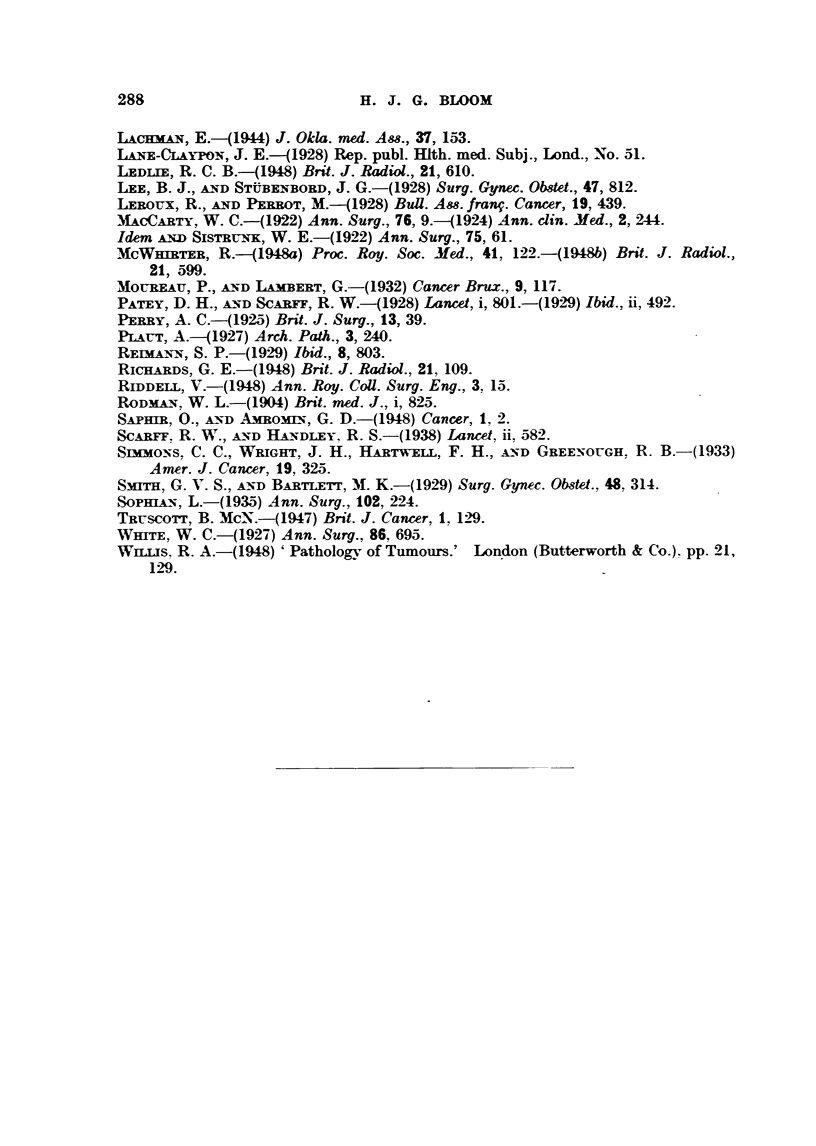# Prognosis in Carcinoma of the Breast

**DOI:** 10.1038/bjc.1950.26

**Published:** 1950-09

**Authors:** H. J. G. Bloom

## Abstract

**Images:**


VOL. IV SEPTEMBER, 1950 NO. 3

### PROGNOSIS IN CARCINOMA OF THE BREAST.

H. J. G. BLOOM.

*From the Bland-Sutton Institute of Pathology, Middlesex Hospital, London, W.1.*

Received for publication May 18, 1950.

In recent years there has been much controversy concerning the treatment of mammary carcinoma. Not only has the type of therapy most likely to achieve success been the subject of dispute, but also the same measures in the hands of well-known authorities have produced a wide variation in results. It seems likely that the difficulties arise from comparing the results obtained in groups of cases which are not strictly comparable. Before the different methods can be assessed adequately we must have a reliable system for classifying patients with this disease. The importance of this is two-fold. Firstly, it provides a more accurate guide to prognosis, and secondly, it ensures the proper grouping of similar cases for comparison. Have we a classification which accomplishes this ?

The outlook for a patient with cancer of the breast depends first and foremost upon the degree of malignancy of the tumour and upon its extent. It is a wide belief that the prognosis is also influenced by certain other features, but the value of these is open to question and will form the subject of a separate inquiry. Suffice here to state that such factors as age of patient, site of tumour in the breast, size of primary growth and duration of symptoms were not found, *per se*, to influence the outlook materially.

How can we determine the degree of malignancy of mammary cancer and also its extent ? The former problem will be discussed presently. The latter is assessed by the results of a clinical examination, and forms the basis of classifications of this disease now in general use. These are undoubtedly of value, but they fail to account for at least a 25 or 30 per cent five-year mortality for growths which are apparently confined to the breast (i.e. Stage 1). In addition, there are also surprising successes achieved for the more advanced cases.

Attempts have been made in the past to group patients with breast cancer according to histological criteria which, it has been claimed, give some indication as to the degree of malignancy of the tumour. Two methods are available. One depends upon the relative amounts of epithelial and connective tissue elements, and gives such types as encephaloid, scirrhus and atrophic scirrhus. The other is based upon a system of grading, Whilst the results of the former have largely proved disappointing, conflicting views exist as to the value of the latter. The problem of grading has been investigated chiefly in the United States.

It has received but little attention in this country, though Patey and Scarff (1928) and later Scarff and Handley (1938) pointed out its importance in carcinoma of the breast.

It is the prime object of this paper to investigate the significance of the grade of malignancy in mammary cancer in an attempt to establish a more accurate system of classifying patients with this disease. It is thus hoped to contribute information which will not only assist in formulating prognosis, but also aid in the search for the best form of treatment.

There is great difficulty at the present time in deciding on the line of action to adopt in view of the widely different procedures advocated by different authorities. The magnitude of the problem facing surgeons and radiotherapists can perhaps be appreciated by considering the divergence of opinion expressed during the course of a single year regarding the management of early cases (i.e. Stage 1). Whilst Gordon-Taylor (1948) and Cade (1948) believe radical mastectomy alone to be the most suitable treatment for these patients, Riddell (1948) and also Richards (1948) advocate ancillary radiotherapy. On the other hand, even the value of the radical operation has been challenged. Keynes (1937) was the pioneer in this country of more conservative measures for breast cancer, and now McWhirter (1948*a*, 1948*b*) has complicated the issue further by making a case for the simple operation with deep X-rays to the undissected axilla.

Breast cancer, by virtue of its acccessibility, would appear to be an example of malignant disease ideally suited for surgical treatment. It is now over fifty years since Moore of London conceived and introduced the radical mastectomy (Rodman, 1904), and still there is controversy in some quarters as to its value. If we cannot solve the problems of mammary carcinoma, what hope is there for the surgery of malignant disease in less accessible sites ?

### *Previous Attempts at Grading.*

Von Hansemann in 1893 was the first to point to the relationship between the histological picture and the behaviour of tumours. He introduced the term " anaplasia," which implied a loss of differentiation and the increase of reproductive power of a growth. He concluded in 1902 that, with rare exceptions, the greater the anaplasia, the greater the tendency to form metastases (Plaut, 1927).

Little attention was directed to the grading of tumours until Broders (1920) applied von Hansemann's theory to squamous cell carcinoma of the lip, and a year later to similar tumours of the skin (Broders, 1921). He revealed a close relationship between the degree of anaplasia and the length of survival.

Greenough (1925) was the first to grade cancer of the breast according to the principle of anaplasia. The prognostic value of a number of epithelial features were studied and three grades of malignancy were recognized. A parallel was found between the histological picture and the progress of the patient.

MacCarty and Sistrunk (1922) and MacCarty (1922, 1924) also studied the histology of breast tumours, and found the most important prognostic characteristics to be the degree of cellular differentiation and the presence or absence of certain stromal features.

White (1927) was unable to agree with the views of MacCarty, but in a series of 100 cases confirmed the results of Greenough (1925).

Patey and Scarff (1928), in a small preliminary group of cases, largely followed Greenough's method of classification, and showed a definite correlation between the histology and clinical course in breast cancer. This work was supported by the results of a larger investigation carried out by Scarff and Handley (1938).

Smith and Bartlett (1929), and later Simmons, Wright, Hartwell and Greenough (1933), also confirmed the value of the system introduced by Greenough (1925). The five-year survival rates for the three grades of malignancy obtained by these authors are comparable, and agree in the main with those of Patey and Scarff (1928).

Lee and Stübenbord (1928) considered that too much emphasis had been placed on histological grading. They therefore elaborated a " clinical index of malignancy " based upon age, presence of lactation, rate of growth of tumour, and extent (stage) of the disease. Three groups were established and 100 cases studied. To compare clinical with histological grading Ewing classified the same cases into three grades ; details of his method were not given. The authors concluded that histological grading is less effective in furnishing a prognosis than their clinical plan. Recently Richards (1948) has modified this system and included a factor for site of growth. He claims the method to be the most reliable guide to prognosis available at present. On the other hand, it has not been generally accepted that age and site of tumour are of prognostic significance. Perry (1925), Lane-Claypon (1928), Truscott (1947) and Harnett (1948) were not able to prove that the position of the growth had any bearing on outcome in breast cancer. Similarly, age was not found of value by Haagensen and Stout (1943), Truscott (1947), Harnett (1948) and others. These points, however, will be discussed more fully in a subsequent investigation.

Flothow (1928), in a little over 200 cases of carcinoma of the breast, studied the factors stated by MacCarty (1922) to represent a defensive mechanism by the body to a malignant tumour. His results support the views of MacCarty, and the presence or absence of these factors were considered to be of value in prognosis.

Hueper has gone so far as to devise a " histological malignogram " based upon no less than twenty different features. It was shown that the malignancy increased with a rise in the " malignancy index." For comparison a clinical classification was also elaborated by Schmitz, but no attempt was made to study the effect of correlating these histological and clinical systems (Hueper and Schmitz, 1929).

Opinions regarding the accuracy of histological grading have varied from Hueper, who finds as many as twenty different features of prognostic value (Hueper and Schmitz, 1929), to Reimann (1929), who is unable to find one. The latter, employing a number of histological criteria, tried unsuccessfully to grade 100 cases of breast cancer. He concluded that it is futile to decide from an examination of a section of the tumour what will happen to the patient.

Plaut (1927) reviewed the literature on the grading of cancer, and does not advocate the method of histological prognosis for practical purposes.

More recently Willis (1948) states that attempts at precise numerical histological grading of tumours as an index of their malignancy are very arbitrary, unscientific and wasted effort. He considers, however, the broad grouping methods based on general histological stucture—presumably such as that of Greenough (1925)—to be of value.

*Criteria Employed in Histological Grading.*

The histological features which have been chosen as the basis for grading breast cancer have varied with different authors. While some have attached chief importance to parenchymal features, others have directed their attention to the stroma; many have combined both elements. MacCarty (1922) found cellular differentiation, hyalinization, fibrosis and lymphocytic infiltration to be of prognostic value. His criteria were employed by Flothow (1928), who reached a similar conclusion. Greenough (1925), on the other hand, applying the principal of anaplasia, concentrated on tubule formation, variation in size of cells and nuclei, secretory activity and mitotic and hyperchromatic figures. He found round cell infiltration and hyalinization of little value in estimating prognosis. White (1927) investigated the factors employed by MacCarty (1922) and by Greenough (1925). His results support the views of only the latter.

The principles laid down by Greenough (1925) were closely followed by others, such as Smith and Bartlett (1929), and Simmons, Wright, Hartwell and Greenough (1933). Patey and Scarff (1928) and Scarff and Handley (1938), however, attached chief importance to tubule formation, variation in size of nuclei, and mitotic and hyperchromatic figures.

Hueper (Hueper and Schmitz, 1929) employed many features in both the parenchymal and stromal elements in his complex grading plan.

Delbet and Mendaro (1927) in France, on the other hand, considered the presence or absence of mucous secretion to be the most important factor in ascertaining the outcome of patients with mammary carcinoma. They used the mucicarmine stain to study this feature. Leroux and Perrot (1928) and Moureau and Lambert (1932) support the views of Delbet and Mendaro (1927), but Betrand and de Nagey (1931) consider muco-secretion to be of no value in determining clinical progress.

Because of the conflicting views regarding the prognostic significance of various histological features in cancer of the breast, Haagensen (1933) investigated 15 different factors separately. They fall into three main groups—manner of growth, cell morphology and reaction of stroma. The author based a grading system on those elements he found to be of prognostic value. These were papillary arrangement, comedo arrangement, adenoid arrangement, variation in size and shape of nuclei, number of mitoses and gelatinous degeneration. Three grades of malignancy were recognized, and were found to bear a close relationship to the period of survival. Haagensen (1933) concluded that histological grading is simply an additional prognostic guide, and should be subordinate to the clinical data.

Evans (1933) studied a number of parenchymal and stromal features, and concluded that, although anaplasia indicates a poor prognosis, it is of no practical value in assessing the outcome for the individual case. His results largely agree with those of Greenough (1925), but number of mitoses was found to be of no significance.

Sophian (1935) also investigated both parenchymal and stromal factors in breast carcinoma. The most important criteria appeared to be adenoid arrangement, constancy of nuclear size, actual nuclear size, mitoses and secretion. Three grades of malignancy were established, and a definite correlation was found between histology and prognosis.

Harrington (1937), in a clinical and pathological study, graded a large series of breast cancers according to the method of Broders (1920). He concluded that the grade of malignancy is the most important indication ot survival period, and this is more significant when considered together with the state of the axillary glands.

From what has been said it is clear that opinions vary regarding the prognostic value of histological grading. Even when a close parallel was demonstrated between microscopic appearance and clinical progress, some workers have hesitated to advocate the method for practical purposes.

Complicated, exact numerical systems, such as the "histological malignogram" introduced by Hueper (Hueper and Schmitz, 1929), are time-consuming and of doubtful value.

Most success in the field of histological grading for breast cancer appears to be with the method of Greenough (1925). This has been employed by several authors, and found to be of definite value in indicating the likely outcome of patients with this disease.

### PRESENT INVESTIGATION.

*Material.*

The series taken for the present investigation consists of 565 cases of carcinoma of the breast treated at the Middlesex Hospital between the years 1936 and 1942 inclusive, and at the war sector units associated with it during the latter half of this period. In spite of the movement of population produced by the recent conflict, all but 11 of the cases were traced by the follow-up department of the hospital. Success, however, was not so great in collecting the histological slides, and in 36 cases they could not be obtained. There were 12 post-operative deaths and 4 air-raid casualties. These 63 cases were excluded, as were a further 32 for various reasons tabulated below. With these deductions 470 patients were left for study.

| | Number of cases. |
|---|---|
| Total | 565 |
| To be excluded— | |
| Untraced | 11 |
| Post-operative deaths | 12 |
| Air-raid casualties | 4 |
| No histological sections available | 36 |
| No carcinoma or query carcinoma in sections available | 23 |
| Impossible to grade | 7 |
| Sarcoma | 2 |
| | 95 |
| Remaining for consideration | 470 |

It is to be noted that in seven instances grading was not carried out. In these cases the only sections of tissue available were either too small or else prepared by the frozen technique. Grading in such circumstances, it will be pointed out later, is apt to be inaccurate.

Of the total cases, 209 were treated by surgery alone, and 261 by surgery with ancillary radiotherapy. Further details of treatment will be considered later. It must be emphasized that all patients in this investigation were subjected to some form of surgical procedure. Pure radiotherapy cases could not be considered owing to the absence of biopsy or surgical specimens for grading purposes. It is therefore obvious that the most advanced and hopeless cases have been excluded. Hence, our results are likely to be superior to those of previous reports from this hospital in which these cases were taken into account.

For the present study the five-year survival rate will be the chief prognostic yard-stick, though some attention will also be given to ten-year results. "Survival rate," however, does not necessarily imply freedom from cancer; it is merely an indication of the number of patients actually alive. In point of fact, many of them have obvious, distant secondary deposits.

*Method of grading.*

The method of grading used by Patey and Scarff (1928) was employed. This was based on the principles laid down by Greenough (1925), but chief importance was attached to tubule formation, regularity in size, shape and staining of nuclei and hyperchromatism and mitoses.

*Tubule formation.*—Well-marked tubule formation was considered of favourable prognosis. This view is supported by the work of Greenough (1925), White (1927), Patey and Scarff (1928), Smith and Bartlett (1929), Sophian (1935), and Scarff and Handley (1938). In fact, this is the only histological feature on which practically all workers in this field have agreed is of prognostic value.

*Regularity in size, shape and staining of nuclei.*—The greater the irregularity the worse the prognosis. This was found to be the case by the same group of workers quoted in the previous paragraph. Certain authors, such as Evans (1933), Greenough (1925) and Haagensen (1933), paid attention to variation in size of the cells, but the outlines of these are usually indistinct; it is more reliable to consider the nuclei. This feature is considered one of the best indications of the degree of anaplasia, and therefore of the malignancy of the growth.

*Hyperchromatic nuclei and mitoses.*—The greater the number present the more gloomy the prognosis. Evans (1933) was unable to support this view, and Plaut (1927) considers the feature unreliable on the grounds that many cancer cells divide by amitosis. Nevertheless, as Haagensen (1933) points out, a considerable proportion of the cells in cancer of the breast divide by mitosis, and he found a close relationship between the number present and the outcome. This is also supported by the work of Greenough (1925), White (1927), Patey and Scarff (1928), Smith and Bartlett (1929), Sophian (1935), and Simmons, Wright, Hartwell and Greenough (1933).

It was noted whether each of the above three factors was absent or present in slight, moderate or marked degree. The tumour was then placed in one of the following grades:

Grade I. Carcinoma of low malignancy.
Grade II. Carcinoma of moderate malignancy.
Grade III. Carcinoma of high malignancy.

Microphotographs illustrating examples of these grades are given in Fig. 1 to 12.

The cases were fairly evenly distributed, 30 per cent of the total being placed in Grade I, 41 per cent in Grade II, and 29 per cent in Grade III. Similar results were obtained by Greenough (1925), Haagensen (1933), and Scarff and Handley (1938). White (1927), however, placed most of his cases in the intermediate grade. Harrington (1937), on the other hand, using Broder's (1920) method, found the majority of his material falling into the highest grade of malignancy, 51 per cent in Grade IV compared with only 4 per cent in Grade I.

### *Grading in the Present Series.*

*Grade and prognosis.*

When grading of the cases in the present investigation had been completed the clinical details were obtained from the follow-up department of the hospital. The five-year survival rate for all treatments was determined for each grade of tumour; these are shown in Table I.

TABLE I.—*Grade and Prognosis. 5-Year Survivals (All Treatments).*

| Grade. | Cases. | Alive at 5 years. No. | Alive at 5 years. Per cent. |
|---|---|---|---|
| I | 141 | 111 | 79 |
| II | 191 | 81 | 42 |
| III | 138 | 35 | 25 |
| Total | 470 | 227 | 48 |

It is evident that a parallel exists between grading and prognosis, there being more than three times the number of patients alive with Grade I than with Grade III tumours.

The present results may be compared with those of other authors who have employed Greenough's (1925) principles in grading (Table II). It will be noted that they agree especially with the findings of Patey and Scarff (1928), and

TABLE II.—*Grade and Prognosis by Various Authors.*

| Grade. | 5-year survivals according to author. Greenough, 1925. Per cent. | Patey and Scarff, 1928. Per cent. | White, 1927. Per cent. | Smith and Bartlett, 1929. Per cent. | Simmons *et al.*, 1933. Per cent. | Scarff and Handley, 1938. Per cent. |
|---|---|---|---|---|---|---|
| I | 68 | 69 | 66 | 87 | 83 | 45 |
| II | 33 | 42 | 47 | 42 | 45 | 29 |
| III | *Nil* | 23 | *Nil* | 14 | 25 | 23 |

Simmons, Wright, Hartwell and Greenough (1933). No Grade III cases remained alive in the series of Greenough (1925), and also of White (1927), but this does not appear to be the general experience.

The total survival rate of 48 per cent obtained here agrees very closely with the results of certain other writers in much larger investigations. Thus, 48 per cent was also given by Harrington (1946) among some 5700 patients at the Mayo Clinic. Richards (1948) quotes 43 per cent for a little over a thousand cases, and Adair (1943), 51 per cent for a similar number.

The mean duration of life in months for cases dying within ten years of operation is shown in Table III. Once again a parallel is demonstrated between grade of malignancy and duration of life, Grade I patients living twice as long as those belonging to Grade III.

TABLE III.—*Mean Duration of Life for Cases Dying within Ten years of Operation.*

| Grade. | Cases dead. | Survival in months. |
|---|---|---|
| I | 41 | 59·6 |
| II | 103 | 40·3 |
| III | 74 | 30·1 |
| Total | 218 | 43·3 |

The five-year survival period is a convenient yard-stick with which to gauge prognosis in breast cancer, but as Truscott (1947) and others have pointed out, a large number of fatalities from this disease occur from five to ten years after operation. Hence, surgeons are now becoming more and more interested in longer follow-up periods. For this reason the ten-year results will also be given here. Three hundred and ten cases, treated between the years 1936 and 1939 inclusive, were available for this study. The survival rates for the three grades of malignancy are displayed in Table IV. As with the shorter follow-up a parallel

TABLE IV.—*Grade and Prognosis. 10-Year Survivals (All Treatments)*

| Grade. | Total cases. | Alive at 10 years. | |
|---|---|---|---|
| | | No. | Per cent. |
| I | 82 | 41 | 45 |
| II | 144 | 41 | 28 |
| III | 84 | 11 | 13 |
| Total | 310 | 93 | 30 |

### EXPLANATION OF PLATES.

FIG. 1.—Low malignancy (Grade I). Well-marked tubule formation ; nuclei regular in size, outline and staining ; absence of hyperchromatic nuclei and mitotic figures. × 45.

FIG. 2.—As for Fig. 1. × 350.

FIG. 3.—Low malignancy (Grade I). Well-marked tubule formation ; nuclei regular in size, outline and staining ; occasional mitotic figures. × 45.

FIG. 4.—As for Fig. 3. × 350.

FIG. 5.—Moderate malignancy (Grade II). Moderate tubule formation ; moderate irregularity in size, outline and staining of nuclei ; moderate number of hyperchromatic nuclei and mitotic-figures. × 45.

FIG. 6.—As for Fig. 5. × 350.

FIG. 7.—Moderate malignancy (Grade II). Slight attempt at tubule formation ; moderate irregularity in size, outline and staining of nuclei ; few mitotic figures. × 45.

FIG. 8.—As for Fig. 7. × 350.

FIG. 9.—High malignancy (Grade III). No tubule formation ; moderate irregularity in size, outline and staining of nuclei ; numerous hyperchromatic nuclei and mitotic figures. × 45.

FIG. 10.—As for Fig. 9. × 350.

FIG. 11. High malignancy (Grade III). No tubule formation ; marked irregularity in size, outline and staining of nuclei ; numerous hyperchromatic nuclei and mitotic figures. × 45.

FIG. 12.—As for Fig. 11. × 350.

Bloom.

7

8

9

10

11

12

is again revealed between grade and prognosis, there being between three and four times the number of cases alive with Grade I than with Grade III tumours.

Comparison of the five- and ten-year results emphasizes the importance of the latter as a more accurate indicator of the control of mammary carcinoma, there being a striking fall in the numbers of survivals for the corresponding grades of tumour, even with allowance for expected deaths from other causes.

The ten-year survival rate for all patients was 30 per cent. This is in keeping with the 26 per cent obtained by Richards (1948), and the 32 per cent by Harrington (1946).

*Prognostic value of the individual factors employed in grading.*

The prognostic importance of each of the three factors employed in grading was investigated separately. The results are set out in Table V. It is seen that well-marked tubule formation is of value in indicating a relatively good prognosis. There appears to be little difference in outcome among the cases with moderate and those with slight or no tubule formation. Variation in size of nuclei and number of hyperchromatic and mitotic figures appear to be of greater value in grading. These results agree on the whole with those of Greenough (1925) and Haagensen (1933), who studied their grading factors separately.

TABLE V.—*Individual Grading Factors and Prognosis.*

| Factor. | Degree. | Cases. | 5-year survivals. Per cent. |
|---|---|---|---|
| Tubule formation | (i) Well-marked | 105 | 73 |
| | (ii) Moderate | 256 | 42 |
| | (iii) Slight or *nil* | 109 | 39 |
| | Total | 470 | 48 |
| Variation in nuclei | (i) Slight | 87 | 72 |
| | (ii) Moderate | 225 | 53 |
| | (iii) Marked | 158 | 29 |
| | Total | 470 | 48 |
| Hyperchromatic and mitotic figures | (i) Very few or *nil* | 73 | 86 |
| | (ii) Moderate | 231 | 52 |
| | (iii) Marked | 166 | 27 |
| | Total | 470 | 48 |

*Grade, glandular involvement and prognosis.*

As various authors have pointed out, it is unwise to rely on grading alone for the assessment of prognosis. The extent of the growth should also be taken into account, and the most important single factor indicating this is whether the axillary lymph nodes are histologically involved or not. In the present material these structures were available for examination in 382 instances. The five-year survivals for cases with and without this complication are seen in Table VI. It is evident that more than twice the number of patients without glandular spread are alive compared with those whose glands are invaded. This proportion agrees very closely with the one given by Harrington (1937) in over 4000 cases at the Mayo Clinic.

TABLE VI.—*Glandular Involvement (Histological) and Prognosis.*

| Axillary glands. | Cases. | 5-year survivals. No. | 5-year survivals. Per cent. |
|---|---|---|---|
| Not invaded | 170 | 121 | 71 |
| Invaded | 212 | 68 | 32 |
| Total | 382 | 189 | 52 |

The results of combining histological grading with the state of the axillary nodes are shown in Table VII. This procedure clearly enhances the value of grading. The outlook for patients with Grade I tumours without axillary metastases is very different than for those with Grade III growths and this complication (94 per cent. alive at five years in the former group compared with 16 per cent in the latter). It is particularly interesting to note that the prognosis in the lower grade of malignancy with glandular spread is better than for either the higher or intermediate grades without such metastases (65 per cent alive at five years compared with 53 per cent and 61 per cent respectively).

TABLE VII.—*Glandular Involvement, Grade and Prognosis. (All Treatments).*

| Axillary glands. | Grade. | Cases. | 5-year survivals. No. | 5-year survivals. Per cent. |
|---|---|---|---|---|
| Not invaded | I | 62 | 58 | 94 |
| | II | 74 | 45 | 61 |
| | III | 34 | 18 | 53 |
| Total | | 170 | 121 | 71 |
| Invaded | I | 43 | 28 | 65 |
| | II | 92 | 28 | 30 |
| | III | 77 | 12 | 16 |
| Total | | 212 | 68 | 32 |

These results agree in principle with those of most workers who have employed the system introduced by Greenough (1925), and are almost identical with the findings of Simmons, Wright, Hartwell and Greenough (1933) (Table VIII). Patey and Scarff (1928) and Scarff and Handley (1938), on the other hand, found a uniformly good result in all three grades when the glands were not involved, and so concluded that grading is only of prognostic value in those cases with axillary metastases. The balance of evidence, however, does not appear to support this view.

TABLE VIII.—*Glandular Involvement, Grade and Prognosis* (*Simmons, Wright, Hartwell and Greenough*, 1933).

| Axillary glands. | Grade. | 5-year survivals. Per cent. |
|---|---|---|
| Not invaded | I | 92 |
| | II | 62 |
| | III | 50 |
| Invaded | I | 65 |
| | II | 32 |
| | III | 20 |

*Relation between histology and glandular metastases.*

A parallel has been demonstrated between the histological appearance of breast cancer and prognosis. Can the histology be correlated more directly with the tendency of the growth to metastasize ? With reference to this question Table IX has been constructed. It is evident from a study of this table that the proportion of cases with involvement of the axillary lymph nodes increases with the rise in grade of malignancy. The significance of this, however, cannot be accurately assessed without considering the time factor. Thus any type of breast cancer will give rise to axillary spread, provided that it is present in the patient for sufficient length of time. The figures in Table IX refer to the state of the cases at the time of operation, and the time factor involved here is the delay in seeking treatment. This delay was assumed originally to be fairly comparable for each group of patients. On the other hand, if for any reason the Grade III cases tend to present for treatment after a longer interval of time than those belonging to Grade I, then this may account for the greater incidence of axillary involvement in the former. Investigations, however, revealed that this was not the case. On the contrary, the patients with tumours of higher grade malignancy appear to have symptoms of briefer duration as revealed in Tables X, XI.

TABLE IX.—*Cases with Axillary Involvement According to Grade of Malignancy*

| Grade. | Cases. | With axillary involvement. | |
|---|---|---|---|
| | | No. | Per cent. |
| I | 105 | 43 | 41 |
| II | 166 | 92 | 55 |
| III | 111 | 77 | 69 |
| Total | 382 | 212 | 55 |

TABLE X.—*Mean Duration of Symptoms According to Grade.*

| Grade. | Cases. | Mean duration of symptoms. Months. |
|---|---|---|
| I | 124 | 10·1 |
| II | 174 | 7·6 |
| III | 125 | 7·1 |
| Total | 423 | 8·3 |

TABLE XI.—*Distribution of Cases According to Grade and Duration of Symptoms.*

| Grade. | Duration of symptoms in months. 6 or less. Cases per cent. | 7–12. Cases per cent. | More than 12. Cases per cent. | Total. Per cent. |
|---|---|---|---|---|
| I | 56 | 21 | 23 | 100 |
| II | 70 | 18 | 12 | 100 |
| III | 69 | 21 | 10 | 100 |

It is evident that the mean duration of symptoms is shorter for women with Grade III than with Grade I growths. In addition a higher percentage of the former present within six months of the onset of the disease and fewer after twelve months as compared with the Grade I cases. Further attention to this feature will be given in a subsequent paper. Suffice at present to employ the information solely for the purpose of lending weight to the conclusions implied in Table IX. Thus, in spite of the shorter delay in seeking treatment, there is a greater incidence of axillary involvement among the high and intermediate grades of malignancy, consequently emphasizing the greater metastasizing power of these types of growth.

The evidence presented here points to the fact that the degree of malignancy of breast cancer is reflected in its histology. Ewing (1940), however, prefers to employ the term "potential malignancy" for a neoplasm as determined by microscopic examination, because the clinical course may be modified by such features as site of tumour, surgical trauma and perhaps changes in rate of growth. He concludes that with these limitations a close parallel exists between histological structure and malignancy.

### *Clinical Staging for Prognosis.*

Several authors, such as Hueper and Schmitz (1929), and also Lee and Stübenbord (1928), have compared the results of grading and staging in breast cancer. Opinions have varied as to which procedure offers the most reliable guide to prognosis. Let us, therefore, examine the effects of staging in the present series and compare the results with those of the histological classification.

### *Staging.*

Records for assessing the stage which the growth had reached were available in 466 instances. The Manchester Plan was employed, but glandular involvement was determined, where possible, by microscopic examination. The reason for this will be pointed out later.

*Clinical staging system (Manchester).*

Stage 1.—The growth is confined to the breast. Involvement of the skin directly over and in continuity with the tumour does not affect staging provided that the area involved is small in relation to the size of the breast.

Stage 2.—As for Stage 1, but there are palpable mobile lymph nodes in the axilla.

Stage 3.—The growth is extending beyond the corpus mammae, as shown by—
- (a) The skin is invaded or fixed over an area large in relation to the size of the breast.
- (b) The tumour is fixed to underlying muscle. Axillary glands may or may not be palpable, but if glands are present they must be mobile.

Stage 4.—The growth has spread beyond the breast area, as shown by—
- (a) Fixation of axillary glands indicating extension outside the capsule.
- (b) The tumour is completely fixed to the chest wall.
- (c) Secondary deposits in the supra-clavicular lymph glands.
- (d) Secondary deposits in the skin wide of the tumour.
- (e) Secondary deposits in the opposite breast.
- (f) Distant metastases, e.g. bone, liver, brain, lung, etc.

The five-year survivals according to stage are shown in Table XII. For comparison the ten-year results are also given (Table XIII). As previously, when the cases were classified according to grade (Tables I, IV), a large number of deaths are seen to take place in the interval between the shorter and longer follow-up periods, thus once more emphasizing the importance of the latter in assessing the achievements of breast cancer therapy.

A study of grading and also staging indicates that both methods give comparable results ; the latter, however, has the slight advantage of revealing the outcome for the most advanced cases (Stage 4).

TABLE XII.—*Stage and Prognosis. Five-year Survivals (All Treatments).*

| Stage. | Cases. | Alive at 5 years. | |
|---|---|---|---|
| | | No. | Per cent. |
| 1 | 156 | 115 | 74 |
| 2 | 145 | 61 | 42 |
| 3 | 126 | 45 | 36 |
| 4 | 39 | 5 | 13 |
| Total | 466 | 226 | 48 |

TABLE XIII.—*Stage and Prognosis. Ten-year Survivals (All Treatments).*

| Stage. | Cases. | Alive at 10 years. | |
|---|---|---|---|
| | | No. | Per cent. |
| 1 | 109 | 52 | 48 |
| 2 | 83 | 20 | 24 |
| 3 | 86 | 20 | 23 |
| 4 | 29 | 0 | 0 |
| Total | 307 | 92 | 30 |

*Relation between histology and stage.*

We have already drawn attention to the relationship in breast cancer between the histological architecture of the tumour and its tendency to involve the axillary glands (Table IX). A further opportunity is now present to correlate grading with invasive and metastasizing power for this type of growth.

The distribution of the cases according to the stage which the neoplasm has reached is shown for each histological grade (Table XIV). Exactly half of the Grade I tumours have not yet extended beyond the first stage, whilst about a quarter are advanced, belonging to Stages 3 or 4 taken together. Roughly the reverse occurs for the cases of high malignancy. Intermediate figures are present for the intervening Grade II.

TABLE XIV.—*Distribution of Cases in Each Grade According to Stage.*

| Grade. | Percentage of cases in each stage. | | | Per cent total. |
|---|---|---|---|---|
| | 1. | 2. | 3 + 4. | |
| I | 50 | 23 | 27 | 100 |
| II | 31 | 34 | 35 | 100 |
| III | 20 | 36 | 44 | 100 |

These results merely give additional support to the view already expressed with regard to the relation between histology and axillary involvement, that the higher the grade of mammary carcinoma, the greater the tendency for early spread beyond the breast.

*Stage and Grade for Prognosis.*

It has been shown under the previous heading that there is little to choose between a method of grading and staging in determining prognosis for mammary carcinoma. The vast majority of workers in this field, however, have argued as to the relative merits of either system. But why should the grade and stage be considered only separately, and in the sense of competing with each other to form the basis of a reliable prognostic guide ? A combination of these features would result in a classification which gives information on not only the type of growth, but also its extent when first seen. Would this not bring about a more accurate grouping of cases than could be produced by either system alone ? Indeed, several authors have taken the histological picture into consideration with the state of the axillary lymph nodes, and the value of this has been confirmed here (Table VII). On the other hand, a survey of the literature appears to indicate that a combined system of grading and more complete staging has yet to be presented.

The possible value of this will now be examined, the results being shown in Table XV and emphasized in Fig. 13. The percentage of five-year survivors is seen to range from as high as 87 per cent for Stage 1, Grade I cases down to as low as 6 per cent for those belonging to Stage 4, Grade III. It is also evident that whilst the prognosis in each stage deteriorates with the rise in histological malignancy, the cases with low grade tumours do not conform to the general trend. The outlook for these cases is better in Stage 2 than it is for the intermediate or high grades in the first stage. Furthermore, it is really remarkable that the low grade cases in Stage 3 have a better prognosis than either the intermediate or high grades in the second and even the first stages. Only a few cases are present in Stage 4, but even here the results are seen to be in accordance with those in the earlier categories.

TABLE XV.—*Stage, Grade and Prognosis. Five-year Survivals (All Treatments).*

| Stage. | Grade. | Cases. | 5-year survivals. No. | 5-year survivals. Per cent. |
|---|---|---|---|---|
| 1 | I | 70 | 61 | 87 |
| | II | 58 | 39 | 67 |
| | III | 28 | 15 | 54 |
| 2 | I | 31 | 22 | 71 |
| | II | 64 | 26 | 41 |
| | III | 50 | 13 | 26 |
| 3 | I | 36 | 25 | 69 |
| | II | 47 | 14 | 30 |
| | III | 43 | 6 | 14 |
| 4 | I | 2 | 2 | 100 |
| | II | 20 | 2 | 10 |
| | III | 17 | 1 | 6 |
| | Total | 466 | 226 | 48 |

FIG. 13.—Stage, grade and prognosis. Percentage of cases alive at 5 years (all treatments).

From these results the value of linking grading with staging is at once clear. Such a procedure gives a far more accurate indication of prognosis than either system alone. It reveals that all is by no means lost if a patient presents with a Stage 2 or even a State 3 growth provided that it belongs to the lowest grade of malignancy (Grade 1). It also explains why certain cases with Stage 1 tumours give disappointing results, and why some in Stage 3 do surprisingly well.

The ten-year survivals are also given for the 307 cases treated between the years 1936–1937 inclusive (Table XVI, Fig. 14). These are seen to be uniformly less than those for the shorter five-year follow-up, the same pattern of results, however, being maintained.

TABLE XVI.—*Stage, Grade and Prognosis. Ten-year Survivals (All Treatments.)*

| Stage. | Grade. | Cases. | 10-year survivals. | |
|---|---|---|---|---|
| | | | No. | Per cent. |
| 1 | I | 42 | 23 | 55 |
| | II | 50 | 22 | 44 |
| | III | 17 | 7 | 41 |
| 2 | I | 14 | 6 | 43 |
| | II | 37 | 11 | 30 |
| | III | 32 | 3 | 9 |
| 3 | I | 25 | 12 | 48 |
| | II | 39 | 7 | 18 |
| | III | 22 | 1 | 5 |
| 4 | I | 0 | 0 | 0 |
| | II | 16 | 0 | 0 |
| | III | 13 | 0 | 0 |
| | Total | 307 | 92 | 30 |

FIG. 14.—Stage, grade and prognosis. Percentage of cases alive at 10 years (all treatments).

### *Comparison of Two Series of Cases of Breast Cancer.*

Having revealed the value of combining histological grading with clinical staging for prognosis in mammary carcinoma, the suggestion is now put forward that the wide range in results produced by different authors for identical methods of treatment depends upon the comparison of incomparable groups of cases. In all probability a similar explanation also accounts for the confusion which exists as to the relative merits of the various lines of therapy advanced for this disease.

The fallacy in grouping cases is considered to result from employing a system of classification based solely upon either the histological or the clinical picture. What evidence is there to support these views ?

With reference to this question, two groups of cases will be taken for study, one treated by surgery alone, and the other by surgery with ancillary radiotherapy. Although it is not the purpose of this paper to discuss the merits of treatment in breast cancer, the results achieved by these two methods will be compared in order to illustrate the discussion.

*Method of treatment.*

Of the 470 cases in the present series, 209 were treated by surgical measures alone, and 261 by the combined method of surgery and irradiation. These will form the two groups for study. It must be pointed out that the treatment in each group was by no means uniform, there being considerable variation both from the surgical as well as the radiological aspect. In the purely surgical cases, however, the vast majority (180) had a radical mastectomy. In the radio-surgical group there were 206 examples of this operation. Irradiation was predominantly in the form of deep X-rays, either pre-operative, post-operative, or both, a few cases being treated with radium alone or radium and deep X-rays.

The surgical treatment for all but two cases was carried out at the Middlesex Hospital or the Sector Hospitals associated with it during the recent war. The radiotherapy was administered by the Meyerstein Institute of the Middlesex Hospital.

*Results of treatment.*

*According to grade.*—Let us first consider the results achieved for each group according to the grade of malignancy (Tables XVII, XVIII). It is evident that the survival rates obtained by surgery alone appear to be superior to those of the combined treatment for all three classes of tumour.

TABLE XVII.—*Grade and Prognosis—Surgical Cases.*

| Grade. | Cases. | Alive at 5 years. | |
|---|---|---|---|
| | | No. | Per cent. |
| I | 79 | 67 | 85 |
| II | 85 | 44 | 52 |
| III | 45 | 14 | 31 |
| Total | 209 | 125 | 60 |

TABLE XVIII.—*Grade and Prognosis—Radio-surgical Cases.*

| Grade. | Cases. | Alive at 5 years. No. | Per cent. |
|---|---|---|---|
| I | 62 | 44 | 71 |
| II | 106 | 37 | 35 |
| III | 93 | 21 | 23 |
| Total | 261 | 102 | 39 |

*According to stage.*—The next step will be to study the results of treatment with regard to the clinical stage instead of the histological grade (Tables XIX, XX). Here again the largely superior results of surgery are obvious. These findings agree closely with those of Harnett (1948) in a larger series of cases (Table XXI).

TABLE XIX.—*Stage and Prognosis—Surgical Cases.*

| Stage. | Cases. | Alive at 5 years. No. | Per cent |
|---|---|---|---|
| 1 | 102 | 77 | 75 |
| 2 | 57 | 29 | 51 |
| 3 | 43 | 19 | 44 |
| 4 | 7 | 0 | 0 |
| Total | 209 | 125 | 60 |

TABLE XX.—*Stage and Prognosis—Radio-surgical Cases.*

| Stage. | Cases. | Alive at 5 years. No. | Per cent. |
|---|---|---|---|
| 1 | 54 | 38 | 70 |
| 2 | 88 | 32 | 36 |
| 3 | 83 | 26 | 31 |
| 4 | 32 | 5 | 16 |
| Total | 257 | 101 | 39 |

TABLE XXI.—*Stage, Treatment and Prognosis* (*Harnett*, 1948).

| Stage. | Alive at 5 years. Radical operation. Per cent. | Radical and radiotherapy. Per cent. |
|---|---|---|
| 1 | 68 | 64 |
| 2 | 44 | 37 |
| 3 | 34 | 32 |
| 4 | 0 | 21 |
| Total | 48 | 40 |

Comparison of Tables XVII and XVIII with Tables XIX and XX reveals comparable results for both grading and staging systems. The latter, however, shows the prognosis for the most advanced cases.

*Possible reasons for the inferior results of the radio-surgical cases.*

*Variation in treatment.*—In both series of cases the exact mode of treatment showed much variation. The vast majority of the surgical patients, however, had a radical mastectomy. On the other hand, in the radio-surgical group, although this operation was performed in the majority, there were many instances of more conservative surgery, which included 37 simple mastectomies and 17 local excisions. Because of these facts it may be argued that the variation in treatment produced incomparable groups of cases. Thus the examples of non-radical surgery may, for purposes of comparison, throw an unfair disadvantage on the group receiving ancillary radiotherapy. Any conclusions drawn as to the superiority of surgical over radio-surgical treatment may, therefore, be erroneous.

With this in view, the survivals of a more uniform type of therapy were taken for study, the comparison being made between patients treated by radical surgery alone and radical surgery with irradiation (Tables XXII, XXIII). The results of the former treatment, however, are still seen to be superior to those of the latter. In fact they are almost identical to those given previously for the groups in which the radical operation was not universal (Tables XVII, XVIII). Similar results were obtained when the cases were taken according to stage.

TABLE XXII.—*Results of Radical Mastectomy alone, According to Grade.*

| Grade. | Cases. | 5-year survivals. No. | 5-year survivals. Per cent. |
|---|---|---|---|
| I | 66 | 59 | 89 |
| II | 77 | 40 | 52 |
| III | 37 | 14 | 38 |
| Total | 180 | 113 | 63 |

TABLE XXIII.—*Results of Radical Mastectomy and Radiotherapy, According to Grade.*

| Grade. | Cases. | 5-year survivals. No. | 5-year survivals. Per cent. |
|---|---|---|---|
| I | 31 | 21 | 68 |
| II | 78 | 27 | 35 |
| III | 60 | 15 | 25 |
| Total | 169 | 63 | 37 |

The greater achievement of surgery alone over the combined attack is again seen in Harnett's (1948) figures which are also for the radical operation and are classified by stages (Table XXI).

From these facts it is clear that the variation in treatment by surgery in the present material does not appear to interfere with the fair comparison of the surgical and radio-surgical groups of cases, and cannot be called upon to account for the inferior results of the latter.

*Distribution of cases.*—The application of either a grading or a staging system separately for the classification of patients with breast cancer may result in fallacious grouping.

Let us first of all consider a system of grading. In this instance a false impression may be given when the results of two series of cases are compared owing to the possible unequal distribution of clinical stages in corresponding histological grades. Thus, in any given grade one of the series may have more early and fewer advanced growths than the other. In fact this has been found to be the case in the present investigation.

Reference to Table XXIV and Fig. 15 shows the distribution of the early and later stages in the three grades of malignancy according to treatment. To simplify the picture Stage 2 has been omitted from the histogram and Stages 3 and 4 taken together. It is evident that in each class of tumour there are more early and fewer advanced cases in the series treated by surgery alone than by the combined method.

TABLE XXIV.—*Distribution of Cases in Each Grade According to Stage and Treatment.*

| Grade. | Stage. | Surgical cases. | | Radio-surgical cases. | |
|---|---|---|---|---|---|
| | | No. | Per cent. | No. | Per cent. |
| I | 1 | 50 | 63 | 20 | 33 |
| | 2 | 16 | 21 | 15 | 25 |
| | 3 | 13 | 16 | 23 | 39 |
| | 4 | 0 | 0 | 2 | 3 |
| Total | | 79 | 100 | 60 | 100 |
| II | 1 | 36 | 42 | 22 | 21 |
| | 2 | 28 | 33 | 36 | 35 |
| | 3 | 18 | 21 | 29 | 28 |
| | 4 | 3 | 4 | 17 | 16 |
| Total | | 85 | 100 | 104 | 100 |
| III | 1 | 16 | 36 | 12 | 13 |
| | 2 | 13 | 29 | 37 | 40 |
| | 3 | 12 | 26 | 31 | 33 |
| | 4 | 4 | 9 | 13 | 14 |
| Total | | 45 | 100 | 93 | 100 |

Total cases, 466

Our next step is to consider staging. What of the distribution of histological grades of malignancy in the various clinical stages ? Reference to Table XXV and Fig. 16 shows a similar arrangement to the above. For the sake of simplicity

FIG. 15.—Distribution of early and advanced cases according to grade and treatment.
□ Surgery cases. ■ Surgery—radiotherapy cases.

only Grades I and III are shown in Fig. 16. Stage 4 is omitted owing to the small number of cases subjected to surgery alone in this group. Here there are seen to be more low and fewer high grade tumours in each stage of the surgical as compared with the radio-surgical cases. Furthermore, of all the neoplasms of low grade malignancy (Grade I) 57 per cent were in this surgical group in contrast to only 30 per cent of the highly malignant type (Grade III) (Table XXVI). Hence, as with grading, so staging should not be employed as the sole factor in comparing the results of different treatments.

It is well known that the more advanced cases are referred for radiotherapy, and this accounts for the distribution shown in Fig. 15. What has not been previously clear is that the cases in a corresponding stage for both treatments may not be strictly comparable. In the present series more are of high and fewer of low grade malignancy in the group receiving radiotherapy (Fig. 16). The clinician is unaware of the histology of the tumour he examines before considering treatment. What, then, may account for this fact ? Is it merely fortuitous ? Chance, it is true, may play a part, but this is not the only possibility.

Although two groups of patients are placed in the same clinical stage, there may be some difference in the extent of the growth for which no allowance is made in our present staging systems. For example, the number of glands involved in Stage 2 and the degree and extent of attachment in the third stage are variable features. The surgeon is probably influenced by such matters to send the more advanced cases of any particular stage for ancillary radiotherapy. In view of the parallel revealed between histological picture and extent of tumour (Tables IX, XIV), it is these unfavourable cases which also tend to be of high grade malignancy, hence the distribution in Fig. 16.

Here we have investigated two series of cases, each subjected to a different line of therapy. The same problems of classification, nevertheless, are applicable to cases treated by identical methods. For example, in the corresponding stages

TABLE XXV.—*Distribution of Cases in Each Stage According to Grade and Treatment.*

| Stage. | Grade. | Surgical cases. No. | Surgical cases. Per cent. | Radio-surgical cases. No. | Radio-surgical cases. Per cent. |
|---|---|---|---|---|---|
| 1 | I | 50 | 49 | 20 | 37 |
| | II | 36 | 35 | 22 | 41 |
| | III | 16 | 16 | 12 | 22 |
| Total | | 102 | 100 | 54 | 100 |
| 2 | I | 16 | 28 | 15 | 17 |
| | II | 28 | 49 | 36 | 41 |
| | III | 13 | 23 | 37 | 42 |
| Total | | 57 | 100 | 88 | 100 |
| 3 | I | 13 | 30 | 23 | 28 |
| | II | 18 | 42 | 29 | 35 |
| | III | 12 | 28 | 31 | 37 |
| Total | | 43 | 100 | 83 | 100 |
| 4 | I | 0 | 0 | 2 | 6 |
| | II | 3 | 43 | 17 | 53 |
| | III | 4 | 57 | 13 | 41 |
| Total | | 7 | 100 | 32 | 100 |

Total cases 466.

FIG. 16.—Distribution of cases of low and high grade malignancy according to stage and treatment.
□ Surgery cases. ■ Surgery—radiotherapy cases.

TABLE XXVI.—*Distribution of Cases According to Grade and Treatment.*

| Grade. | Surgical series. Per cent. | Radio-surgical series. Per cent. | Total. Per cent. |
|---|---|---|---|
| I | 57 | 43 | 100 |
| II | 45 | 55 | 100 |
| III | 30 | 70 | 100 |

of two or more groups of cases, there may be a widely differing number of tumours of high grade malignancy. It is, therefore, useless to compare results obtained by different authors if the cases are taken according to stage and no allowance is made for type of growth. In this instance chance is probably the chief factor accounting for the distribution of the tumours. A process of selection, however, cannot be ruled out, individual surgeons having, to some extent, personal criteria for deciding on the measures to be adopted for a given case.

*Neglected routes of extension.*—Reference will be made to avenues of extension of breast cancer which are largely neglected in both prognosis and also treatment. These are via the internal mammary chain and the supra-clavicular glands. It is likely that the tumours of high grade malignancy tend to spread early via such pathways in the same way as they involve the axillary lymph nodes. Of these unfavourable Grade III cases nearly 70 per cent (Table XXVI) were referred for irradiation, thus emphasizing the disadvantage placed on the radiotherapy group when it comes to the comparison of treatment results.

To sum up : It is postulated that in a given stage certain cases have unfavourable clinical features, which influence the surgeon to refer them for radiotherapy. The tumours of these patients tend to be of high grade histological malignancy, and have probably also extended via routes, as yet, not given adequate consideration in prognosis and treatment. This may account, at least to some extent, for the unfavourable results achieved here and elsewhere (Adair, 1943 ; Truscott, 1947 ; Harnett, 1948) by ancillary radiotherapy. Careful selection of cases is, therefore, probably responsible for the superior results of surgical measures alone.

On the other hand, a number of authors report better results for the combined treatment. In these instances the achievement of radiotherapy is probably even greater than appears on first examination owing to the unfavourable features which tend to be present in cases referred for this treatment. Alternatively the arrangement of the cases may be more comparable than in the present investigation.

It has been pointed out that the problem of grouping also applies to series treated by identical methods, though here chance rather than selection is probably a more important factor accounting for the distribution of the patients.

*Stage, grade, treatment and prognosis.*

By linking staging with grading a more accurate system for determining prognosis has been introduced. With this method it is hoped that further light may be thrown on some of the problems of breast cancer, and possibly the true merits of the various treatments of the disease assessed.

When this clinico-pathological system is applied here, will the results achieved by ancillary radiotherapy appear to be any better ? This question, unfortunately, cannot be answered for the present because the cases available for such a study were too few in number. Thus the 209 surgical and the 261 radio-surgical patients were each divided into twelve groups (four clinical stages, each containing three histological grades). Consequently the numbers in each were too small to be significant for purposes of comparison. Further work is under way to collect a larger series to enable this to be undertaken.

*Treatment and Prognosis.*

A paper on prognosis in mammary carcinoma cannot be complete without reference to the effect of treatment. As already pointed out, it is not our intention to undertake a detailed study at this time into the merits of the different modes of therapy. This problem, which requires a larger series of cases than is at present available, will form the subject of a separate inquiry. Suffice here to indicate some of the points to be investigated.

First of all, does treatment prolong life ? Several authors have published figures on the natural duration of untreated breast cancer. The five-year survival rate appears to be about 20 per cent. Harries (personal communication), at the Middlesex Hospital, quotes 19 per cent for the five-year follow-up and 4 per cent for a period of ten years among some 220 cases. These figures are inferior to those obtained by treatment, which is universally accepted as being of definite value in prolonging life, except perhaps in the very advanced cases.

The next problem which arises is whether the type of treatment influences the outlook. It is here that the controversy begins. How radical should surgery be ? Of what value is ancillary radiotherapy ? Such questions as these are still far from being settled. We have shown that in all probability the dispute arises from comparing groups of cases which are not strictly comparable.

Once the cases are classified more accurately, such as on the lines indicated earlier in this paper, and only then, shall we be in a position to study the merits of various treatments. We may find, for example, that less radical surgery may be adequate for the control of certain cases such as those with Grade I, Stage 1 tumours, whilst radiotherapy may be an important ancillary measure for some cases, but on the other hand of little use for others.

From time to time authors have claimed good results for less radical surgical procedures in breast cancer. Grace (1937) in America obtained a 56 per cent five-year survival rate among 40 cases treated by simple mastectomy and radiotherapy. This author considers that " the cellular structure of the tumour is the dominant factor in prognosis, and surgical technique, irrespective of its radicalism, plays a definite secondary role." Some support for this view is perhaps to be found in the paragraphs which follow.

In the present series 37 cases were treated by simple mastectomy and radiotherapy, and 41 per cent were alive at the end of five years. This agrees with Harnett's figure of 40 per cent in a group of 100 similar cases (Harnett, 1948).

Good results have even been claimed for local excision. This operation, followed by irradiation, was performed in 17 patients of the present investigation, and 65 per cent survived five years. Here again agreement is found with Harnett (1948). A group of 45 cases in his series were treated by local excision with implantation of radium, and 63 per cent were alive after a similar period of time.

Among a large number of cases reviewed by Adair (1943), there were 98 examples of the simple operation and 72 of local excision, the majority being given ancillary irradiation. The survival rate in the former group was 62 per cent, and in the latter 67 per cent.

What do these results mean ? Can it be possible that more conservative surgical measures than the radical operation are sufficient for the control of mammary cancer ? Such, indeed, is the view in this country of Keynes (1937), and more recently McWhirter (1948*a*, 1948*b*).

We have already suggested that when cases are properly grouped according to both stage and grade it may be found that less radical surgery may be adequate for certain types of case. Thus, of the 17 cases in the present series which were treated by local excision and radiotherapy with a survival rate of 65 per cent, 12 belonged to Stage 1, and 10 of these had Grade I growths. Of these 10, 90 per cent were alive at the end of five years. The good results in this group probably depend upon the early stage and low malignancy of the tumours.

We conclude this section by emphasizing that the treatment most likely to achieve success in the control of breast cancer cannot be assessed at the present time. This will become possible only when cases are classified more accurately, such as on the clinico-pathological basis indicated here.

### *Fallacies in Grading and Staging.*

We have advocated a system of classifying bıeast cancer based upon the grade and stage of the growth. Let us now examine these two features for any possible fallacies.

*Grading.*

*Histology of primary growth.*—The most frequent objection to grading as an index to prognosis in mammary carcinoma is that sections cut from different parts of the same tumour may show marked histological variation. This may be true in some cases, but its importance has been over-emphasized. In spite of variability a definite microscopic pattern can be recognized for the vast majority of neoplasms of this type. It is not even necessary to have numerous sections to appreciate this picture ; one or two portions of tissue of reasonable size taken from the periphery of the growth are adequate. However, in those cases where there is much variability in appearance, the most malignant part of the section is considered.

To test these views the following simple investigation was undertaken : Two sections were cut from different parts of the periphery of each of 25 consecutive breast cancers. The 50 slides thus obtained were labelled with a code number and then mixed. Grading was performed, the code of the accompanying twin to each section being unknown to the examiner. It was found that the pair of sections of each breast had been placed in the same grade of malignancy.

Two further points on the histology of the primary neoplasm require attention. Firstly, grading of cancer in minute fragments of tissue is unreliable, and should not be attempted. Secondly, frozen sections are difficult to assess, and where possible, grading should be confined to tumours prepared by the paraffin technique.

*Histology of metastases.*—Should the degree of histological malignancy of the metastases vary considerably from the primary tumour, a false impression as to prognosis may be gained if only the growth in the breast is studied. For example, a tumour of low malignancy may give rise to metastases in which a change of biological character takes place. If the deposits become more malignant then the prognosis will probably be altered. In point of fact, there is reason to believe that an alteration to greater malignancy occurs only on very rare occasions. Thus, Patey and Scarff (1929), in a series of 110 cases, found that the metatases in the axillary nodes were of the same grade as the primary in 82 per cent, of a lower grade in 16 per cent, and of a higher grade in only 2 per cent. Since a considerable proportion of cases tend to show axillary deposits of lower malignancy than the primary growth, grading should be confined to the latter.

*Staging.*

*Axillary glands.*—The importance of considering the extent of the growth in grading has already been pointed out. The state of the axillary glands is usually taken as the arbitrary standard for determining whether the growth is confined to the breast or not. This feature is usually assessed clinically in a staging system. It is unsafe, however, to rely on this form of examination for glandular involvement. Non-palpable glands may show histological invasion, and conversely, palpable glands may exhibit only inflammatory changes. A recent statistical survey of breast cancer (Harnett, 1948) revealed 39 per cent of clinically free glands to be invaded microscopically. In 19 per cent of cases with clinical glandular involvement the enlargement proved to be non-malignant. Comparable figures to these are also given by other writers, such as Harrington (1935), Lachman (1944), Riddell (1948) and Ledlie (1948). Greater accuracy in prognosis will therefore be achieved if glandular invasion is determined, where possible, histologically. This has been the rule in the present series.

Saphir and Amromin (1948), on the other hand, have shown that even routine histological examination of the axillary nodes is open to error. By serial sections they discovered involvement of these structures in 33 per cent of a series of cases previously reported as free from metastases.

*Internal mammary glands.*—Other routes of spread for breast cancer have been largely neglected both in prognosis and treatment. One of the chief pathways is via the internal mammary chain of glands. Handley (1922, 1927) was the first to stress the importance of this avenue of extension, and advocated implantation of radium in the intercostal spaces at the time of operation. Little attention appears to have been given to this problem until Scarff and Handley (1938) suggested that disappointing results in Stage 1 cases may be explained by this mode of spread. It was left, however, to Handley and Thackray (1947, 1949) to provide histological evidence on this point. These authors have shown that extension via the internal mammary nodes may occur early and not infrequently in the absence of axillary metastases. This applies particularly to growths situated in the inner half of the breast ; of 17 such cases 12 had deposits in the internal mammary chain, in 2 of which the axilla was not also involved. In spite of this, Truscott (1947) and Harnett (1948) were unable to show that the site of the growth plays an important part in prognosis.

*Supraclavicular glands.*—A further mode of spread which has not received adequate consideration is via the supraclavicular glands. Halsted, as long ago as 1892, was the first to practise removal of these structures in the radical treatment of cancer of the breast (Rodman, 1904). Twelve years later Rodman (1904) advocated this procedure in those instances where the glands are palpably enlarged, or the topmost axillary nodes show macroscopic involvement. This problem appears to have been subsequently largely neglected. Recently, however, interest has been revived by the work of Andreassen and Dahl-Iversen (1949). These authors removed the supraclavicular nodes as part of the radical mastectomy, and found microscopic involvement in 17 per cent of cases. With invasion of the axilla this figure increased to 33 per cent. If the axilla was free from growth, the supraclavicular nodes did not appear to be invaded. The writers concluded that dissection of the supraclavicular region should be performed if there is extension to the axilla.

In those cases where the axilla appears to be free from involvement at the time of operation and the prognosis subsequently turns out to be poor, invasion has probably occurred by routes such as the internal mammary chain, and perhaps the supraclavicular glands. An attempt to assess the state of the latter is made clinically, but involvement may be microscopic and these structures are not removed as a routine. The state of the internal mammary nodes is usually entirely neglected. These channels of extension may account for at least some of the 13 per cent mortality for Stage 1, Grade I cases (Table XV).

After considering these facts it would appear, as Handley and Thackray (1949) point out, that there are but few growths which are really confined to the breast. It is therefore perhaps surprising to find such good results for even what appear to be early tumours of low malignancy. In the present investigation 94 per cent of Grade I cases without axillary node involvement were alive at five years (Table VII).

### CONCLUSIONS.

A classification for patients with breast cancer which depends upon either a system of staging or grading tends to group cases that are not strictly similar. Comparative studies of such groups is, therefore, apt to be misleading. Unfortunately, methods based upon staging alone are in general use at the present time, and as long as they continue to be employed, so it is likely that there will be no accurate assessment of the various lines of treatment.

Several attempts have been made in the past to establish a more reliable prognostic system, and consequently a better classification, for cases of mammary carcinoma. Chief success, from the point of accuracy and simplicity, appears to have been from histological grading, on the lines indicated by Patey and Scarff (1928), combined with the state of the axillary glands. The value of this method has been confirmed here.

A still more useful method of determining prognosis by linking staging and grading has been presented. Wider adoption of this plan may clarify the position regarding the relative merits of the various treatments advanced for breast cancer. It may even be possible to assess the mode of therapy most likely to achieve the best results for certain groups of cases.

There are two reasons why grading is of importance in prognosis. Firstly, the histological appearance of the tumour is closely related to its " potential malignancy " (i.e. the tendency to spread and form metastases). Secondly, it provides some measure of the probable extent of the growth when the patient is first seen, the most malignant tumours having spread furthest. This latter feature compensates to some degree for those pathways of invasion which are not adequately considered in our present staging systems.

From what has been said, it is evident that the morbid histologist can contribute important information towards the problems of mammary carcinoma. It would appear, however, that this fact has been largely neglected by those concerned with its treatment. Why has there always been at least a 25 per cent five-year mortality for the most favourable cases, i.e. Stage 1 ? This question has defeated explanation in the past, and will probably continue to do so until such time as the pathologist plays a part in establishing a prognostic system of the type indicated in this paper.

Finally, a plea is made for more attention to histological grading in carcinoma of the breast, and a closer co-operation between clinician and pathologist. It may well be that grading of cancer in other sites, on the principles employed here, will be found of similar value.

### SUMMARY.

The controversy which exists as to the relative merits of the different methods of treatment for carcinoma of the breast has led to the present investigation, the prime object of which has been to establish a more accurate classification for this disease.

Previous attempts at histological grading in breast cancer have been reviewed.

Grading has been studied in a series of 470 cases of mammary carcinoma. It has been accomplished without technical difficulty, and a close parallel was found between the histological picture and the period of survival.

The results of grading have been compared with those of clinical staging. Both methods, when employed separately, give comparable results.

The grade of the tumour taken together with the state of the axillary lymph nodes gives a more accurate indication of outcome than either grading or staging alone. For example, 94 per cent of Grade I cases without glandular involvement were alive at five years compared with 16 per cent of Grade III with this complication.

A clinico-pathological system for determining prognosis by linking stage and grade has been presented. This procedure casts light on some of the hitherto unexplained clinical problems of breast cancer. It offers an explanation for disasters in early cases, and also for remarkably good results obtained in advanced stages. In indicates that all is not necessarily lost if a patient presents with a Stage 2 or even a Stage 3 growth, provided it is of low grade histological malignancy.

The five-year results achieved by surgical treatment both alone and in association with radiotherapy have been presented. Inferior results were obtained for the combined attack.

It has been shown that in a given clinical stage or histological grade the cases treated by surgery alone are not strictly comparable to those subjected to surgery and radiotherapy. The latter were found to have additional unfavourable features. This fact has been invoked to explain the disappointing results of ancillary radiotherapy, case selection probably accounting for the superior results of surgical treatment alone.

A classification of breast cancer based upon either a system of grading or staging separately brings about a grouping of cases which are not strictly similar in type. The comparison of such atypical groups may well be responsible for the wide variation in results obtained by different workers for identical methods of treatment. In addition this has probably also played a major part in bringing about the present-day confusion as to the relative merits of the various measures advanced for the control of the disease.

Attention has been drawn to the possible prognostic and therapeutic importance of lymphatic spread to other glands than those in the axilla.

Possible fallacies in grading and staging have been discussed.

Brief reference has been made to the effects of treatment on prognosis. The good results of conservative surgical procedures have been noted.

Note by T. E. Cowan, Esq., F.C.I.S., F.R.S.S.: The interpretations arrived at from the tables shown are statistically sound and call for no special comment.

I am indebted to Professor R. W. Scarff for introducing me to his system of histological grading of breast cancer, and for help and encouragement; to Dr. A. C. Thackray for kind advice and criticism; to Dr. D. K. Sambrook for assessing the clinical stage of the cases; to Miss Chambers of the Follow-up Department for tracing the patients, and to the Photographic Department for assistance with the microphotographs.

For the cases employed in this work my gratitude is due to the surgeons of the Middlesex Hospital and War-time Sector Units, and to Professor B. W. Windeyer, of the Meyerstein Institute of Radiotherapy.

The expenses of this investigation were defrayed by the British Empire Cancer Campaign.

### REFERENCES.

Adair, F. E.—(1943) *J. Amer. med. Ass.*, **121**, 553.

Andreassen, M., and Dahl-Iversen, E.—(1949) *J. int. Chir.*, **9**, 27.

Betrand, I., and de Nagy, A.—(1931) *Pr. méd.*, **39**, 991.

Broders, A. C.—(1920) *J. Amer. med. Ass.*, **74**, 656.—(1921) *Ann. Surg.*, **73**, 141.

Cade, S.—(1948) *Proc. Roy. Soc. Med.*, **41**, 129.

Delbet, P., and Mendaro.—(1927) 'Les Cancers du Sein.' Paris (Masson).

Evans, W. A.—(1933) *Amer. J. Cancer*, **19**, 328.

Ewing, J.—(1940) 'Neoplastic Diseases,' 4th ed. Philadelphia (Saunders), p. 50.

Flothow, P. G.—(1928) *Surg. Gynec. Obstet.*, **46**, 789.

Gordon-Taylor, G.—(1948) *Proc. Roy. Soc. Med.*, **41**, 118.

Grace, E. J.—(1937) *Amer. J. Surg.*, **35**, 512.

Greenough, R. B.—(1925) *J. Cancer Res.*, **9**, 453.

Haagensen, C. D.—(1933) *Amer. J. Cancer*, **19**, 285.

*Idem* and Stout, A. P.—(1943) *Ann. Surg.*, **118**, 859.

Handley, R. S., and Thackray, A. C.—(1947) *Brit. J. Cancer*, **1**, 15.—(1949) *Lancet*, ii, 276.

Handley, W. S.—(1922) 'Cancer of the Breast,' 2nd ed. London (John Murray), pp. 156, 254.—(1927) *Surg. Gynec. Obstet.*, **45**, 721.

Harnett, W. L.—(1948) *Brit. J. Cancer*, **2**, 212.

Harrington, S. W.—(1935) *Surg. Gynec. Obstet.*, **60**, 499.—(1937) *Coll. Pap. Mayo Clin.*, **29**, 668.—(1946) *Surgery*, **19**, 154.

Hueper, W. C., and Schmitz, H.—(1929) *Ann. Surg.*, **90**, 993.

Keynes, G.—(1937) *Brit. med. J.*, ii, 643.

LACHMAN, E.—(1944) *J. Okla. med. Ass.*, **37**, 153.

LANE-CLAYPON, J. E.—(1928) Rep. publ. Hlth. med. Subj., Lond., No. 51.

LEDLIE, R. C. B.—(1948) *Brit. J. Radiol.*, **21**, 610.

LEE, B. J., AND STÜBENBORD, J. G.—(1928) *Surg. Gynec. Obstet.*, **47**, 812.

LEROUX, R., AND PERROT, M.—(1928) *Bull. Ass. franç. Cancer*, **19**, 439.

MACCARTY, W. C.—(1922) *Ann. Surg.*, **76**, 9.—(1924) *Ann. clin. Med.*, **2**, 244.

*Idem* AND SISTRUNK, W. E.—(1922) *Ann. Surg.*, **75**, 61.

MCWHIRTER, R.—(1948*a*) *Proc. Roy. Soc. Med.*, **41**, 122.—(1948*b*) *Brit. J. Radiol.*, **21**, 599.

MOUREAU, P., AND LAMBERT, G.—(1932) *Cancer Brux.*, **9**, 117.

PATEY, D. H., AND SCARFF, R. W.—(1928) *Lancet*, i, 801.—(1929) *Ibid.*, ii, 492.

PERRY, A. C.—(1925) *Brit. J. Surg.*, **13**, 39.

PLAUT, A.—(1927) *Arch. Path.*, **3**, 240.

REIMANN, S. P.—(1929) *Ibid.*, **8**, 803.

RICHARDS, G. E.—(1948) *Brit. J. Radiol.*, **21**, 109.

RIDDELL, V.—(1948) *Ann. Roy. Coll. Surg. Eng.*, **3**, 15.

RODMAN, W. L.—(1904) *Brit. med. J.*, i, 825.

SAPHIR, O., AND AMROMIN, G. D.—(1948) *Cancer*, **1**, 2.

SCARFF, R. W., AND HANDLEY, R. S.—(1938) *Lancet*, ii, 582.

SIMMONS, C. C., WRIGHT, J. H., HARTWELL, F. H., AND GREENOUGH, R. B.—(1933) *Amer. J. Cancer*, **19**, 325.

SMITH, G. V. S., AND BARTLETT, M. K.—(1929) *Surg. Gynec. Obstet.*, **48**, 314.

SOPHIAN, L.—(1935) *Ann. Surg.*, **102**, 224.

TRUSCOTT, B. McN.—(1947) *Brit. J. Cancer*, **1**, 129.

WHITE, W. C.—(1927) *Ann. Surg.*, **86**, 695.

WILLIS, R. A.—(1948) 'Pathology of Tumours.' London (Butterworth & Co.), pp. 21, 129.